# A two stage optimization model for sustainable location routing problem with capacity and time window constraints in smart parcel lockers

**DOI:** 10.1038/s41598-026-41653-6

**Published:** 2026-03-01

**Authors:** S. Mohammad Ghadirpour, S. Kamal Chaharsooghi, Mostafa Hajiaghaei-Keshteli

**Affiliations:** 1 Faculty of Industrial and Systems Engineering, Tarbiat Modares University, Tehran, Iran; 2 Tecnologico de Monterrey, School of Science and Engineering, Monterrey, Mexico

**Keywords:** Smart parcel lockers, Last-mile delivery, Two-stage optimization, Sustainability, Location-routing problem, Metaheuristic algorithms, Engineering, Environmental sciences, Environmental impact

## Abstract

The rapid expansion of e-commerce has significantly increased the demand for sustainable delivery methods to mitigate urban congestion, emissions, and rising logistical costs. Addressing the challenges of last-mile delivery requires logistics providers to respond to operational demands and market dynamics with both urgency and efficiency. Smart parcel lockers, as a known solution, reduce the negative externalities of urban transportation while improving delivery performance. This research introduces a sustainable location-routing model for smart parcel lockers using a two-stage optimization approach. The model aims to minimize operational costs, fuel consumption, and CO₂ emissions while ensuring customer demand is met. Exact solution techniques are applied, and multiple scenarios are evaluated through extensive sensitivity analysis. The model is validated using a real-world case study in Tehran, Iran. In addition, metaheuristic algorithms, such as the Keshtel, Genetic, and Simulated Annealing methods, were benchmarked to evaluate model performance under varying problem scales, with the Keshtel algorithm showing superior scalability and runtime efficiency in large instances. Findings indicate that optimally positioned lockers combined with efficient routing can lead to substantial reductions in both transportation costs and environmental impacts. Practical implications for logistics managers include the integration of electric vehicles and renewable-powered lockers to further advance sustainability goals.

## Introduction

**Fig. 1:**
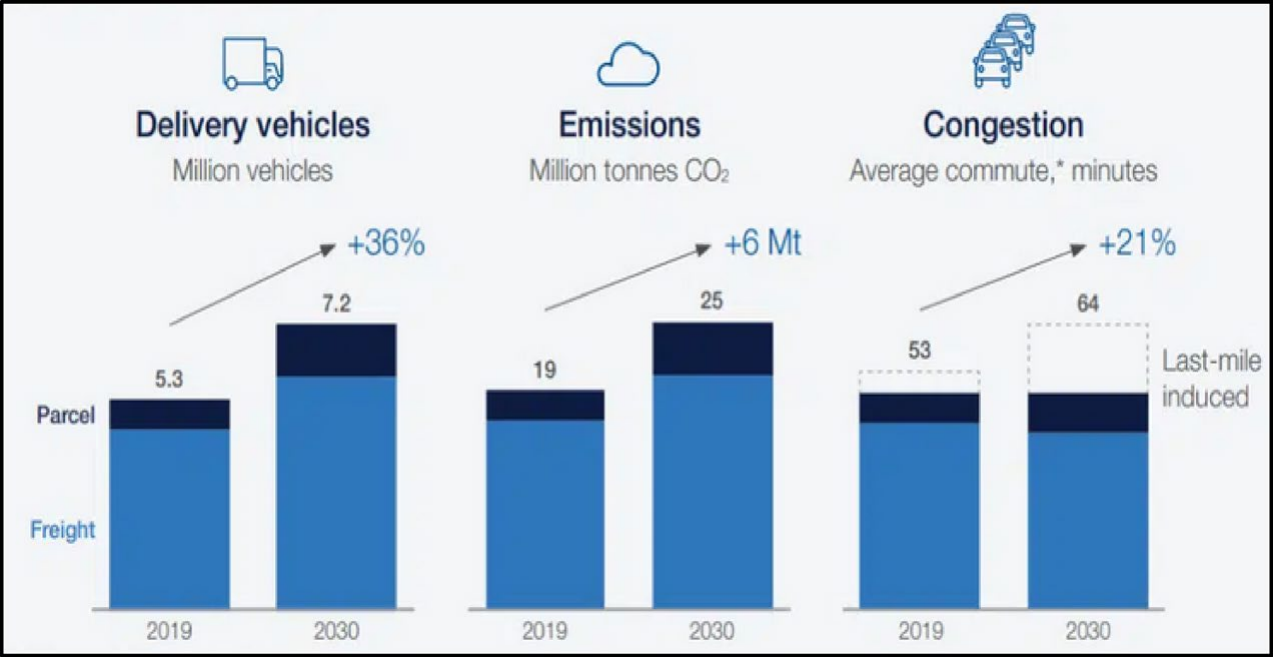
The projected environmental and market impacts of e-commerce logistics by 2030, with anticipated growth in delivery vehicles and CO₂ emissions^13^.

The rapid growth of global e-commerce has significantly altered the retail landscape^1^. The proliferation of online platforms has correspondingly heightened the demand for efficient delivery and logistics services^2,3^. The last-mile delivery phase, which involves transporting goods directly to customers, is particularly challenging^4,5^. It contributes to increased energy consumption, traffic congestion, and CO₂ emissions, especially in urban areas where delivery vehicles navigate densely populated zones^6^. The adoption of green vehicles necessitates adjustments in last-mile distribution systems, driven by their significant influence on the e-commerce market and the growth of on-demand logistics^7^. As depicted in Fig. 1, the environmental impact of e-commerce logistics is expected to worsen by 2030, with a substantial rise in both the number of delivery vehicles and emissions^8^. This trend underscores the urgent need for sustainable solutions in the logistics sector to curb these negative effects^9^. One promising approach to mitigating these challenges is the adoption of smart parcel locker systems^10^. These lockers provide secure, centralized locations where packages can be delivered, allowing customers to retrieve their orders at their convenience^11^. By consolidating deliveries, parcel lockers reduce the need for multiple trips and direct deliveries, leading to savings in energy and a reduction in CO₂ emissions^12^.

**Fig. 2:**
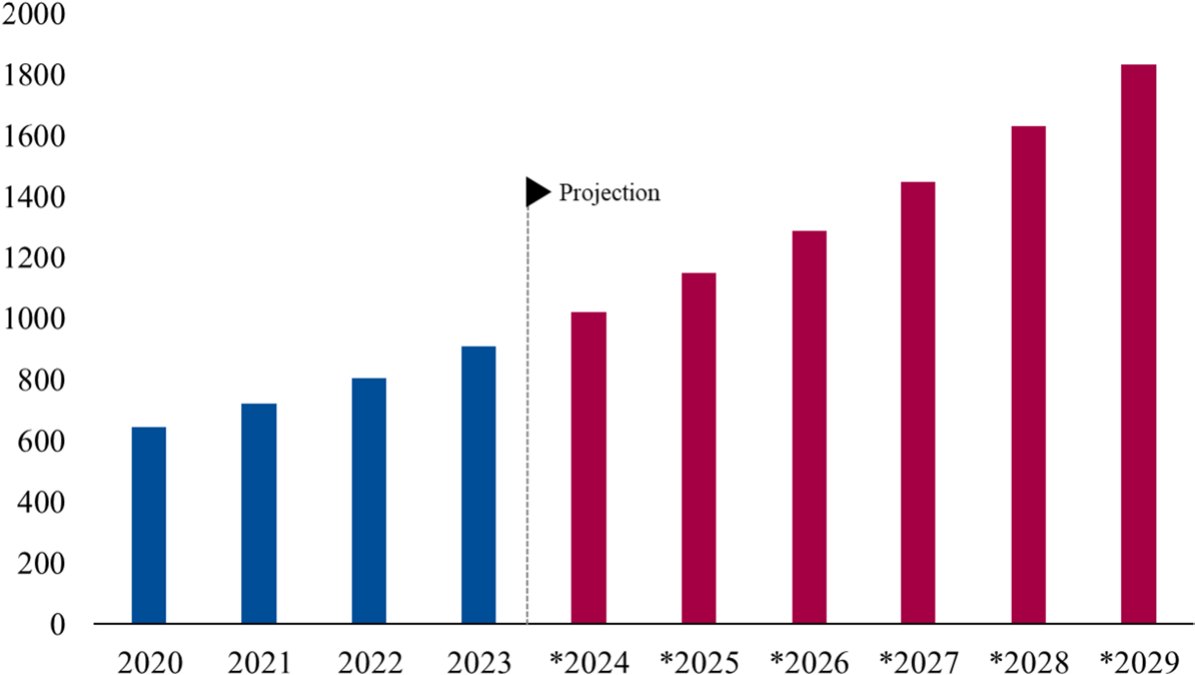
The market size forecast for smart parcel lockers, illustrating steady growth from 2020 to 2030, underscores their increasing role in sustainable logistics solutions^17^.

**Fig. 3:**
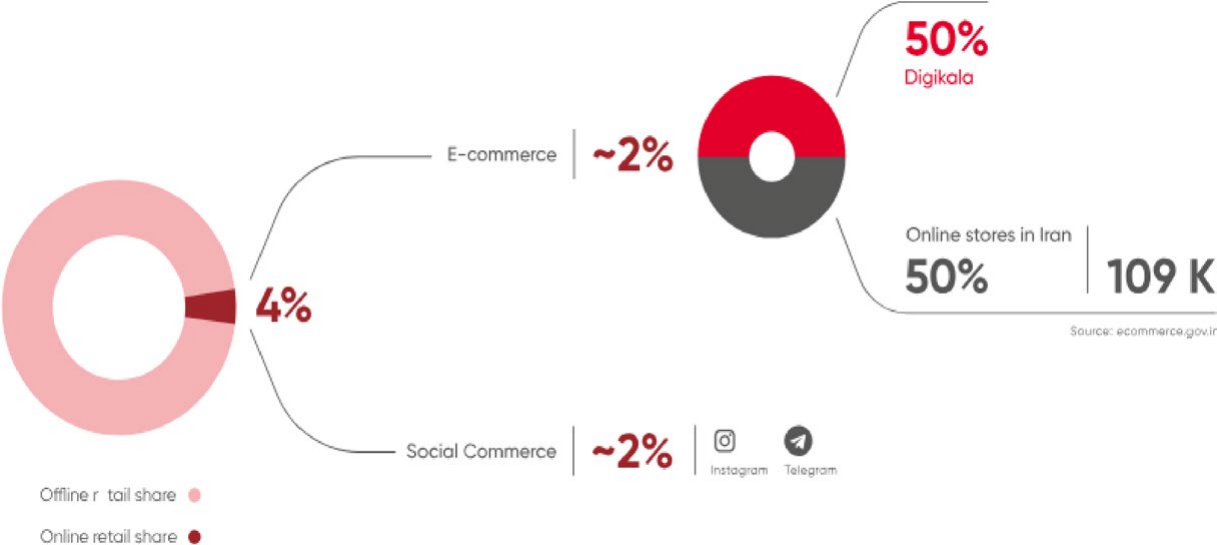
The predominance of offline shopping in Iran’s retail sector, shown in the lower pie chart, highlights the potential for e-commerce expansion, with platforms like Digikala leading the transition towards online shopping^17^.

As shown in the Fig. 2, the market for parcel locker services is projected to grow steadily over the next several years, reflecting their increasing importance as a sustainable logistics solution^14^. Iran, as highlighted in Fig. 3, still has a significant portion of its retail sector based on offline shopping. However, with the rising penetration of online shopping platforms, particularly through popular e-commerce sites like Digikala, there is a strong potential to further expand e-commerce sales. Smart parcel lockers, integrated into this growing e-commerce framework, can play a crucial role in enhancing sustainability by reducing delivery-related traffic and emissions^15^.

Increasing the reliance on online retail could help address traffic congestion and air pollution in major cities, where heavy traffic and environmental degradation are serious concerns^16^.

Given the potential benefits of smart parcel lockers^18^, our research addresses the sustainable location-routing problem for these systems^19^, factoring in capacity and time window constraints. This study adopts a two-stage optimization approach to develop an efficient logistics model. By leveraging mathematical optimization methods and exact solution techniques through GAMS software, we aim to provide insights into the optimal location and routing of smart parcel lockers to maximize efficiency and minimize environmental impact. In addition, the model’s performance is benchmarked against three metaheuristic algorithms to assess its scalability and effectiveness across different problem dimensions.

To consolidate the contribution and positioning of this study, we state the novel elements of the proposed multi-objective, two-stage model and the managerial decisions it enables in sustainable last-mile delivery. Further points summarize the main contributions of the research in a concise manner.

- The study develops a multi-objective, two-stage optimization model that simultaneously determines the optimal placement of smart parcel lockers and the routing of delivery vehicles under capacity and time-window constraints, which creates a unified framework not previously addressed in the regional literature.
- The model incorporates sustainability-driven objectives by integrating CO₂ emissions, travel distance, and service quality metrics, enabling logistics managers to evaluate trade-offs between cost efficiency and environmental performance.
- The benchmarking analysis compares exact and metaheuristic solution methods and identifies the Keshtel Algorithm as a robust and scalable approach for large urban delivery networks, providing practical guidance for real-world implementation.

The structure of this paper is as follows: we begin with a comprehensive literature review on sustainable logistics and parcel locker systems, followed by the methodology section, which details our mathematical model. We then present an in-depth case study to validate our approach, discuss the results, and conclude with a summary of our findings and recommendations for future research. This structure ensures a logical progression from theoretical background to empirical validation and supports the practical application of our findings in real-world urban logistics planning.

## Literature review

The literature on smart parcel lockers is diverse, covering various aspects such as design, optimization, consumer perceptions, and spatial distribution^20^. A comparative review reveals both commonalities and differences across studies, particularly in terms of methodologies, objectives, and regional focuses. Several studies focus on optimizing locker design and location for operational efficiency. For example, a study on smart locker bank design used scenario-based optimization to balance ergonomic and manufacturing costs, ultimately aiming to maximize profit^21^. Their hypothetical study highlights how effective design can enhance profitability by satisfying order scenarios within physical constraints. Similarly, a bilevel programming model was employed to maximize economic efficiency and user satisfaction in Shanghai^18^. By incorporating user satisfaction into the model, their approach underscores the importance of balancing financial and user-centric objectives.

Both studies emphasize profit maximization, yet optimizing for customer experience represents an expanded focus^18^. Location optimization is another prevalent theme^22,23^.

The evaluate of supply-demand matching for smart lockers in Tianjin highlighted that the mismatch between high-demand areas and high-supply areas can lead to resource wastage^22^. On the other hand, a Spherical Fuzzy AHP model was applied in Dublin to optimize location selection under uncertainty, addressing decision-makers’ hesitancy^23^.While both studies target optimal location strategies, first study emphasize spatial inequities, whereas second one focus on mitigating decision-making challenges.

Consumer satisfaction and perceptions are also widely examined, particularly with IoT-enabled lockers and during the COVID-19 pandemic. Consumer satisfaction in China has been analyzed, finding that service reliability, convenience, and fault handling are critical factors^24^. This finding is consistent with research on smart lockers and customer satisfaction in Vietnam, where convenience, security, and reliability were identified as key influences^25^. Additionally, perceived trust significantly outweighs privacy concerns in the U.S. context^26^. The role of smart lockers in mitigating last-mile delivery inefficiencies is another area of focus. A Community Logistics Strategy (CLS) for real-time delivery updates in Hong Kong’s megacities has been proposed, demonstrating the utility of dynamic strategies in densely populated areas^27^.

Similarly, a self-organizing system that uses an auctioning platform to enhance delivery efficiency in the Netherlands has been proposed, thus reducing redundant travel^28^. Spatial analysis of smart lockers also offers insights into how these systems interact with urban environments.

The environmental impact of open parcel lockers on traffic in Austria has been assessed, noting that strategic location can reduce CO2 emissions^29^.

The spatial distribution of smart lockers compared in Tianjin before and after COVID-19, finding that locker deployment shifted towards residential areas^30^. Spatial characteristics in Singapore have been analyzed using the 5Ds Walkability Framework, finding that proximity to amenities and transit enhances locker usage^31^. These studies converge on the point that spatial factors, including accessibility and population density, significantly influence locker performance and environmental outcomes. While prior research has explored various aspects of smart parcel locker systems and last-mile delivery logistics, certain gaps remain unaddressed in the current literature. The summary of related studies is listed in Table 1.

For instance, some studies focus on optimizing locker design for operational efficiency^32^, while some others highlight spatial mismatches between locker supply and demand in urban areas^30^. However, few studies incorporate environmental sustainability in last-mile delivery, especially through multi-objective optimization that balances cost, environmental impact, and service quality.

Furthermore, the literature lacks comprehensive models that account for capacity constraints, and time-window requirements for locker operations, all of which are essential in enhancing urban sustainability. This research addresses these gaps by integrating environmental considerations with an optimization model that simultaneously tackles locker location, and vehicle routing. The primary contributions of this study are summarized as follows:

- Unlike previous studies (e.g^23^.),, which focus mainly on spatial and operational optimization, this research develops a multi-objective model that also minimizes environmental impacts by optimizing route efficiency and locker location.
- Our model uniquely incorporates these constraints, addressing the practical limitations faced in last-mile logistics that are often overlooked in simpler routing models^18^.
- By analyzing multiple scenarios with varied parameters, this study provides insights into the model’s flexibility under different urban and operational conditions, advancing beyond static approaches typically used in existing studies.
- This research not only optimizes last-mile delivery logistics but also contributes to social and environmental goals by reducing urban traffic congestion and CO₂ emissions, fostering a healthier urban environment, and promoting equal access to efficient delivery options for smaller businesses and underserved communities.

These contributions provide a foundation for future research in sustainable and smart last-mile delivery, offering a model that combines cost-effectiveness, environmental responsibility, and adaptability to real-world constraints.

**Table 1:** Summary of the related studies.

Reference	Aim	Methodology	Solving Approach	Case/ Country	Results	Key Findings
^18^	To optimize community locker locations based on economic efficiency and user satisfaction	Bilevel Programming and Genetic Algorithm	Profit Maximization, User Satisfaction	Shanghai, China	Achieved high profits with balanced user satisfaction, influenced by investment budget	Effective optimization balances profitability with user satisfaction in locker location decisions
^21^	To optimize smart locker bank design considering ergonomic and manufacturing costs	Scenario-Based Optimization	Profit Maximization	Hypothetical, U.S.A	Maximized profits by satisfying order scenarios while accounting for physical constraints	Effective smart locker design can balance cost and user convenience, optimizing last-mile delivery profitability
^22^	To evaluate supply-demand matching of smart parcel lockers in residential areas during the pandemic	Analytical Framework and Spatial Assessment	Gini Coefficient, Location Quotient	Tianjin, China	Found oversupply in some areas, with high demand areas typically within 300 m	Spatial mismatch in SPLs can lead to resource wastage and inequity in access, indicating the need for dynamic adjustments
^23^	To optimize parcel locker locations using Spherical Fuzzy AHP	Spherical Fuzzy AHP with Euclidean Distance-Based Aggregation	Location Selection	Dublin, Ireland	Identified optimal locker locations, balancing hesitancy in decision-making	Spherical Fuzzy AHP is effective for location optimization under uncertainty
^24^	To assess consumer satisfaction and perceptions toward IoT-enabled smart lockers in China	Survey and Confirmatory Factor Analysis (CFA)	Regression Analysis	China	Four key factors (excluding price) significantly affect consumer satisfaction	Service reliability, convenience, and fault handling are critical to consumer satisfaction with IoT-based lockers
^25^	To analyze factors influencing senders’ switching intention to smart lockers	Push-Pull-Mooring Framework	Partial Least Squares Structural Equation Modeling	Vietnam	Attractiveness of smart lockers mediates switching intentions from home delivery	Understanding switching intentions can help in better promoting contactless delivery methods
^26^	To explore factors influencing consumers’ adoption of smart lockers from privacy and technology perspectives	Structural Equation Modeling (SEM)	Technology Acceptance and Protection Motivation	U.S. A	Perceived trust outweighs privacy concerns in consumer adoption decisions	Trust in technology-based services strongly influences consumers’ adoption intentions for smart lockers
^27^	To develop a dynamic strategy for immediate parcel delivery in megacities	Community Logistics Strategy Simulation	Real-Time Delivery Update	Hong Kong	CLS model improves efficiency by updating delivery routes in real-time	Dynamic delivery strategies like CLS are essential for high-density cities to meet consumer demands
^28^	To propose a self-organizing system for real-time parcel delivery allocation	Agent-Based Simulation	Auctioning System	Netherlands	System improved delivery efficiency, reducing redundant kilometers traveled	Self-organizing delivery methods offer flexibility and can significantly enhance operational efficiency
^29^	To assess the environmental impact of open parcel lockers on traffic	Impact Assessment with Simulation	Traffic Analysis	Austria	Positive environmental impact observed, with reduced CO2 emissions in certain scenarios	Parcel lockers can positively affect traffic and emissions, but effectiveness depends on strategic location
^30^	To examine spatial distribution of smart lockers before and after COVID-19 in urban areas	Spatial Analysis with Kernel Density Estimation	Nearest Neighbor Analysis	Tianjin, China	Increase in smart lockers post-COVID-19; distribution shifted towards residential areas	COVID-19 prompted an increase in SPLs, with a more clustered distribution pattern in urban areas
^31^	To study spatial characteristics influencing parcel locker performance	Spatial Analysis using 5Ds Walkability Framework	Location-Based Accessibility Analysis	Singapore	Positive correlation between walkability and parcel locker usage	Proximity to amenities and transit enhances parcel locker efficiency, with potential trade-offs in pickup times
^32^	To investigate multiobjective optimization for service area planning of smart lockers	Multiobjective Optimization (NSGA-II)	Taguchi Method, Genetic Algorithm	Taiwan	Favorable solutions obtained for location planning, considering pandemic impacts	Multiobjective optimization effectively supports efficient service area planning for smart lockers
^33^	To explore automated parcel lockers as a solution to last-mile delivery challenges	Multi-Criteria Simulation-Optimization	Mathematical Programming, Simulation	Poznań, Poland	Optimized locker usage based on demand; seasonal variations accounted for	Simulation and optimization can enhance locker network planning, addressing last-mile inefficiencies
^34^	To determine optimal location of movable parcel lockers under stochastic demand	Robust Optimization using Integer Linear Programming	Cost Minimization	Beijing, China	Reduced costs and optimized locker usage under varying demand scenarios	Movable parcel lockers can enhance flexibility in delivery, reducing operational costs with optimized locations
**This study**	To develop a sustainable location-routing model for smart parcel lockers under capacity and time-window constraints.	Two-stage multi-objective optimization and sensitivity analysis.	Exact optimization via GAMS and benchmarking with KA, GA, and SA.	Urban delivery network, Iran.	Identified optimal locker placement and routing strategies with measurable reductions in distance, emissions, and operational cost.	Introduces an integrated framework that links locker location and routing decisions and provides scalable managerial insights supported by comparative algorithmic benchmarking.

## Results

The challenge of achieving sustainable last-mile delivery has become increasingly urgent due to the rise in e-commerce and the corresponding impact on urban traffic, CO₂ emissions, and operational costs. This study tackled these issues by focusing on the optimization of smart parcel lockers as a strategic solution to address the environmental and logistical demands of last-mile delivery. By consolidating deliveries to centralized locker locations, smart parcel lockers can reduce the frequency of direct-to-door deliveries, thus minimizing fuel consumption and emissions. However, efficient locker location and vehicle routing are essential to maximizing these benefits. In this context, our research developed a two-stage optimization model designed to balance the needs of cost-efficiency, service quality, and environmental sustainability, providing a robust approach to improve last-mile delivery networks. The model was implemented in GAMS software and built around several key constraints and parameters, such as locker capacity, vehicle fuel consumption rates, and time window limitations for deliveries. In the first stage, the model determined the optimal number and location of parcel lockers based on customer demand and operational costs. In the second stage, it optimized vehicle routes to minimize travel distances and maximize capacity utilization.

Through sensitivity analysis, we tested four different scenarios by adjusting travel time, distance allowances, and weighting parameters to understand how the network responds under various conditions. This two-stage approach allowed for a flexible, scalable solution that adapts to different operational environments and demand levels, ensuring that the network can respond effectively to fluctuating market demands while maintaining sustainability goals. The results highlight several critical findings for urban logistics and sustainability. The optimized allocation of parcel lockers in high-demand areas not only reduced travel distance, operational costs, and CO₂ emissions but also improved customer service levels by ensuring accessibility to lockers. Additionally, the model demonstrated that combining strategic locker location with efficient vehicle routing could yield a 15–20% reduction in overall fuel consumption, reinforcing the environmental benefits of the approach. The sensitivity analysis revealed that adjusting parameters such as travel time and distance constraints can further enhance efficiency, though these adjustments must be carefully balanced to avoid introducing inefficiencies. Moreover, flexible vehicle routing and resource allocation were shown to be critical for maintaining service levels during peak demand. The model’s performance was further validated through a comprehensive benchmarking analysis against two well-known metaheuristic algorithms. Results demonstrated that the KA offered the most favorable trade-off between solution quality and computation time, especially for large-scale problem instances. This confirms the model’s scalability and practical applicability for complex, real-world urban logistics systems.

Overall, this study provides logistics managers and urban planners with a practical, data-driven framework for integrating smart parcel lockers into last-mile delivery networks, ultimately promoting a more sustainable and efficient approach to urban logistics. This study advances the field of sustainable last-mile delivery by comparing its two-stage optimization approach with recent studies focused on locker location and vehicle routing. Unlike previous models that primarily address either location optimization or routing efficiency in isolation (e.g^24^^7^..,;), our integrated model tackles both simultaneously, optimizing locker location and vehicle routes under capacity and time-window constraints. This holistic approach provides a more realistic and adaptable solution, demonstrating significant reductions in emissions and operational costs compared to single-stage models, while aligning with emerging trends in urban sustainability and e-commerce logistics.

## Discussion

The obtained results facilitate new insights into how smart parcel lockers can aid in enhancing sustainability, operational efficiency, and service quality within last-mile delivery networks. The proposed two-stage optimization model addressed some of the most relevant challenges in the context of locker-based delivery: capacity limitations of lockers, time-window constraints, and the need to reduce carbon emissions in urban areas. Beyond numerical improvements outlined above, the results indicate how spatial demand patterns strongly affect the performance of the lockers. Indeed, the lockers in high-demand regions consistently absorbed larger shares of parcel assignments to show that the effects of demand concentration are defining in shaping the optimal network configuration. The sensitivity analysis allows a better understanding of how variations in travel times and distances do not merely scale the network but reshape the way demand is distributed across lockers. In some scenarios, increased demands at selected lockers revealed the system’s capability of handling larger volumes, pointing out the opportunity to maintain scalability in lockers’ networks provided their design includes enough flexibility. On the other hand, the balanced allocation of vehicles in the scenarios involving an extension of travel allowances underlines the important role of routing adaptability that guarantees efficient use of resources. Although such flexibility is advantageous, the corresponding results also allow one to recognize that an excessive relaxation of constraints can be associated with some forms of inefficiency; this points to the need for careful calibration of the operational policies. Another effective way to efficiently deal with larger scenarios is the use of mechanisms of problem size reduction that minimize the size of the problem to be solved while preserving all the original constraints^35^. Overall, joint interpretation of scenario outcomes and sensitivity trends leads to the conclusion that the effectiveness of the locker network is affected by interplay between spatial demand, routing feasibility, and environmental objectives.

The findings underscore several practical implications for logistics operators and urban planners aiming to improve delivery performance. Lockers located in high-demand areas, such as PL4 and PL5, consistently reduced travel distances and CO2 emissions. This indicates that siting lockers near dense residential clusters, commercial centers, or frequently visited public spaces can create substantial sustainability gains. Optimizing vehicle routes and capacities within the model shows how flexible resource allocation allows operators to handle fluctuating parcel volumes without compromising service quality. This insight is valuable for managers who aim to maintain efficiency under variable demand conditions. Consolidating deliveries to centralized parcel lockers reduces the frequency of door-to-door delivery trips, decreasing traffic congestion and aligning with sustainability objectives in urban environments. The benchmarking findings reinforce that while GAMS can guarantee optimality for small-scale instances, it quickly becomes infeasible as the network grows, which highlights the necessity of scalable metaheuristic approaches. Among the tested heuristics, the KA provided the most reliable balance between runtime and solution accuracy in medium and large scenarios (L1–L3), making it a strong candidate for operational planning tools that support real-world logistics decisions. In tackling sustainability challenges, this study addressed greenhouse gas reduction, congestion management, and the need for flexible delivery services. The adoption of parcel lockers supports these goals by centralizing deliveries, reducing repeated vehicle trips, and maintaining service quality even when customer demand increases. These managerial insights align with broader sustainability strategies and can guide logistics firms in designing more resilient delivery systems.

**Fig. 4:**
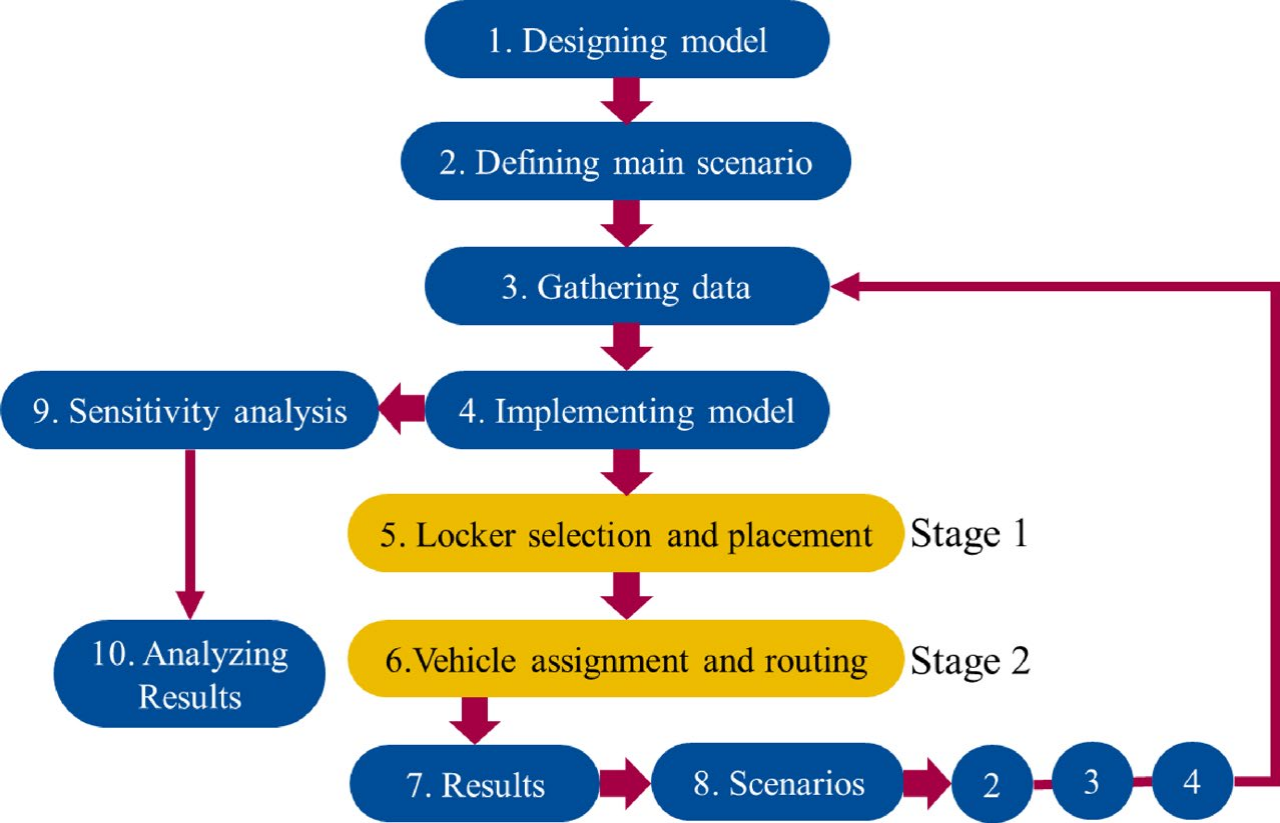
Two-stage optimization model for sustainable last-mile delivery: locker location and vehicle routing with sensitivity analysis.

The structured methodology followed in this study starts with the design of the two-stage model and the definition of the primary scenario, followed by data collection and preparation. The first stage of the model focuses on locker selection and location, determining the optimal number and placement of lockers based on demand distribution and operational costs^36^. his stage addresses the core location-allocation problem and ensures that lockers remain accessible to high-demand areas, which reduces distances traveled, operational expenses, and environmental impacts^37,38^. Figure 4 illustrates the overall workflow of this two-stage optimization process. The second stage involves optimizing vehicle assignment and routing by considering time windows, vehicle capacities, and fuel consumption factors. This results in efficient routes of delivery that further reduce travel distance and emissions. Several scenarios were tested to assess the adaptability of the model in different conditions of the system; sensitivity analysis was then performed as a way to analyze how changes in key parameters have an impact on the outcomes. This two-stage structure, therefore, provides a holistic basis for designing sustainable last-mile delivery systems that can balance operational feasibility with environmental objectives.

**Fig. 5:**
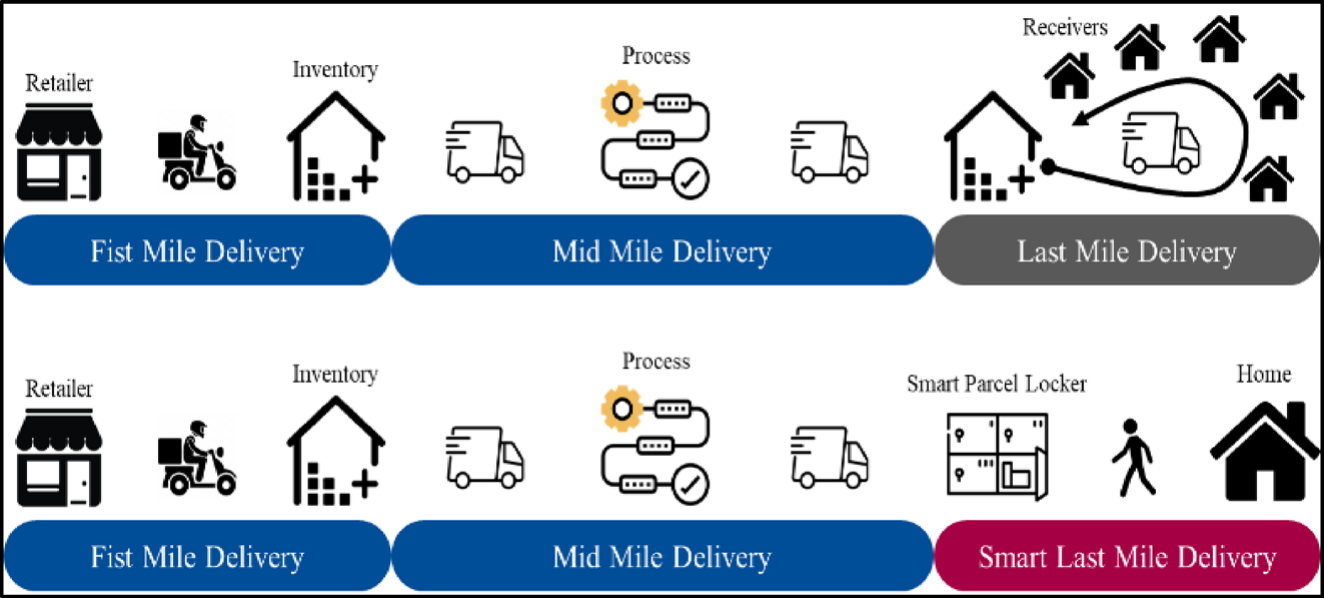
Traditional vs. smart delivery process^40^.

The delivery mechanisms illustrated in Fig. 5 further highlight how smart parcel lockers provide a significant improvement over traditional delivery system. The first scenario presents the conventional three-stage structure of First Mile Delivery, Mid Mile Delivery, and Last Mile Delivery, a structure that often leads to multiple stops, longer routes, and increased fuel consumption. Efficient allocation of transportation equipment enhances the operational resilience of logistics companies and strengthens their ability to manage uncertainties^39^. The second scenario introduces a smart last-mile delivery framework where strategically placed parcel lockers shorten the delivery distance, reduce operational costs, and significantly cut CO2 emissions. This smart system offers a competitive advantage by providing a more sustainable and efficient alternative to conventional delivery approaches.

Although the model offers a strong foundation for optimizing smart parcel locker networks, several limitations present opportunities for future research. The model assumes static demand and does not account for real-time fluctuations, peak delivery periods, or behavioral factors such as customer pickup preferences. This model can be further developed toward adaptive locker network planning by incorporating predictive analytics or real-time demand forecasting. Travel times within the routing stage are considered deterministic; in reality, congestion, delays, and disruptions in cities introduce uncertainty in routing feasibility. Extension to a model that considers stochastic travel times or robust optimization will address this limitation. Additionally, the authors do not yet model customer heterogeneity, for instance, in terms of the customer’s willingness to walk to a locker or his/her preferred time for picking up deliveries. Capturing these behavioral factors would certainly be beneficial to the accuracy of modeling service levels. Finally, this model might also be combined with, for example, multi-echelon delivery systems or electric vehicle routing to expand its applicability to smart and sustainable logistics networks. These extensions will provide decision-makers with resilient, flexible, and environmentally aligned last-mile delivery strategies tailored to dynamic city conditions.

Beyond the operational and environmental benefits, the model carries important socioeconomic implications for Iran’s e-commerce landscape. The current structure of last-mile delivery in Iran is dominated by only a few large logistics providers, bringing forth concentrated market power and limited participation from smaller courier firms and neighborhood-based services. By introducing a network of smart parcel lockers with optimized allocation and routing, the proposed system can lower entry barriers for smaller operators, given that they no longer require extensive urban fleets or high fixed costs to provide competitive services^27^. This redistribution of operational advantage can therefore weaken monopolistic structures and support a more open and diverse logistics market^41^. Such decentralization by way of locker-based delivery will also provide access for local businesses, small retailers, and micro-entrepreneurs to the very same delivery infrastructure as larger firms, further promoting an inclusive e-commerce ecosystem. From the policy point of view, the findings suggest that supportive regulatory frameworks and municipal coordination are crucial in enabling such a system. Cities can ensure fair access to parcel lockers by prioritizing locations in underserved areas, transit hubs, and mixed-income neighborhoods. This will make sure that efficiency gains do not disproportionately benefit high-income districts. Furthermore, the reduction in congestion and emissions will be in tune with Iran’s wider environmental and urban management goals. Encouraging small business participation through subsidies, shared models of locker ownership, or public-private partnerships could further raise the social value of the system. In sum, the proposed model contributes not only to optimization and sustainability but also opens up opportunities for a more inclusive, competitive, and socially responsive logistics sector in Iran.

## Methods

The mathematical modeling is delineated in this section. The mathematical model presented is designed to optimize the allocation of smart parcel lockers and the routing of vehicles for last-mile delivery, focusing on minimizing overall operational costs while adhering to various capacity and time constraints^42^. The model involves several key sets, including nodes representing potential locker locations and the central depot, vehicles in the fleet, and customers^27^. Each locker has a pre-determined capacity, and the vehicles are heterogeneous in terms of fuel consumption rates, capacity, and costs, reflecting real-world complexities in logistics. The aim of the model is to determine the optimal number of lockers to be established, their locations^33^, the vehicle routes^34^, and which customers should be assigned to each locker while minimizing total costs and ensuring that lockers do not exceed capacity.

The objective function minimizes several key factors, including the total costs of establishing lockers, the costs of rejected parcels, vehicle routing costs, and time-related penalties.

Constraints ensure that the lockers serve customers within a maximum allowable distance and that vehicle capacities are not exceeded. Additionally, all lockers must be visited exactly once by a vehicle, and each vehicle must return to the central depot after completing its route. The model uses a scalarization technique to handle multi-objective optimization, applying a weighted sum of different objectives to balance various cost components.

This mathematical approach ensures an efficient delivery system with reduced emissions, optimized routing, and strategic locker location to meet customer demand. The assumptions of the model play a critical role in defining the structure and constraints of the optimization process:

1. The model assumes that all lockers have the same storage capacity, which is predetermined before the optimization process begins. This uniform capacity simplifies the decision-making process and reflects the early stage of planning (referred to as “time zero”). This means that the model does not account for fluctuating locker sizes or dynamic capacity adjustments during operations.
2. Each locker can serve a limited number of customers, and the model ensures that the total demand allocated to any given locker does not exceed its capacity. This constraint is crucial in maintaining service quality and avoiding overloading lockers, which could lead to inefficiencies or operational failures.
3. The model considers a fleet of vehicles that vary in their fuel consumption rates, carrying capacities, and operational costs. These characteristics are predefined and fixed for each vehicle. By incorporating vehicle heterogeneity, the model reflects real-world scenarios where companies use different types of vehicles, each with distinct performance metrics, which must be accounted for in routing and scheduling decisions.
4. The model does not assume a fixed number of lockers from the outset. Instead, it determines the optimal number and locations of lockers as part of the decision-making process. This flexibility allows the model to adjust the network design based on customer demand, cost considerations, and operational constraints.
5. The model allows for the possibility that not all vehicles in the fleet will be utilized. Additionally, each vehicle can only perform one trip, meaning it cannot visit multiple lockers or routes in a single operational cycle. This assumption helps to optimize resource allocation and prevent unnecessary vehicle usage, contributing to cost efficiency.
6. Once a locker is established, it must be visited exactly once by a vehicle during each delivery cycle. This ensures that the deliveries to lockers are completed in a single trip, avoiding redundant visits that could increase operational costs or create inefficiencies.
7. After visiting the designated lockers, each vehicle must return to the central depot. This closed-loop routing ensures that vehicles start and end their routes at the same location, which simplifies the management of fleet operations and ensures consistency in the delivery network.
8. If a vehicle enters a node (such as a locker location or the central depot), it must also leave that node. This ensures that vehicles do not remain stationed at any location indefinitely, and all movements are accounted for within the optimization model.
9. The model assumes that all parcels delivered to the lockers will be collected by customers. There are no scenarios where parcels are left unclaimed, which simplifies the logistics and focuses the model on efficient delivery and routing rather than post-delivery complications.

The modeling takes place at time zero. There is a central depot, represented by node 0, where the process of consolidating each customer’s parcel occurs. Each customer is assumed to have exactly one parcel, meaning the demand for each customer is set to one unit. Customers cannot receive their parcels directly from the central depot. Customers’ usage of lockers may either be accepted or rejected. If accepted, the customer must be assigned to a specific locker; otherwise, the logistics company is required to deliver the parcel to the customer’s doorstep, incurring an additional cost (α). The distance between at least one locker and each customer must be less than the maximum allowable distance. If the total demand of customers within the allowable distance exceeds the capacity of the lockers, then the demand for each locker is equal to its capacity; otherwise, the demand is equal to the total demand of customers located within the allowable distance to that locker. Metaheuristic and exact for locker *l* (d (*l*)) are expressed as Eq. 1:

1 $$\:\sum\limits_{\:\left(j=1|dis\left(l,j\right)<{dis}_{max}\right)}^{m}p\left(j\right)=d\left(l\right);\:\forall\:l$$

The scalarization technique and the Pareto method are two commonly used strategies in multi-objective optimization problems. However, in this specific context, the Pareto method is not suitable for solving the problem, so the scalarization technique is applied instead^43^. Scalarization involves assigning different weights to the objective function terms, which allows for converting a multi-objective optimization problem into a single-objective one by combining the objectives into a weighted sum^32^. This approach enables the decision-maker to prioritize certain objectives over others based on their importance. This ensures that the various objectives, such as minimizing cost, time, or environmental impact, are balanced according to their assigned importance in the overall optimization process. By adjusting these weights, the model can emphasize different aspects of the problem, such as giving more importance to reducing delivery time or minimizing fuel consumption.

**Table:** 

Indices				
*I*		Set of all nodes, indexed by i. *I* = {0, 1, 2, …, $\:i$, …, n}		
*L* ⊆ *I*		Set of potential locker locations, indexed by $\:l$, $\:{l}^{{\prime\:}}$. L = 1, 2, …, $\:l$, $\:{l}^{{\prime\:}}$,…, …, n		
*V*		Fleet of vehicles, indexed by v. V= {1, 2, …, v}		
*J*		Set of customers, indexed by j. J= {1, 2, …,$\:\:j$, …, m}		

***Parameters***				
$\:FCR$		Total objective function		
$\:{O}_{1},\:{O}_{2},\:\dots\:,\:{O}_{6}$		Objective function expressions		
$\:{w}_{1},\:{w}_{2},\:\dots\:,\:{w}_{6}$		Weights related to the O1…O6		
$\:{C}^{f}$		Cost per unit of fuel		
$\:{C}^{FR}\left(i,l,v\right)$		Fuel consumption rate for vehicle v traveling from node i to l		
$\:{C}^{v}\:\left(v\right)$		Cost of utilizing the vehicle v		
$\:f\:\left(l\right)$		The fixed cost of setting up any locker		
$\:\alpha\:$		Penalty cost for rejecting each parcel		
$\:dis\left(l,j\right)$		Distance between node l and customer j		
$\:{dis}_{max}$		Maximum allowable distance between lockers and customers		
$\:{C}^{p}\left(l,j\right)$		Cost of serving customer j from locker l		
$\:d\left(l\right)$		Demand of locker l		
$\:p\left(j\right)$		Demand of customer j		
$\:{q}^{v}\left(v\right)$		Capacity of vehicle v		
$\:{q}^{p}$		Lockers capacity		
$\:t\left(i,l,v\right)$		Travel time from node i to l for vehicle v.		
$\:{t}_{Max}$		Maximum allowable travel time of each vehicle.		
$\:s\left(i\right)$		Service duration at node i.		
$\:\left[{a}_{i},{b}_{i}\right]$		Time window for service at node i.		

***Variables***				
**Variable**	**Type**		**Description**	**First/Second stage decision variable**
$\:{u}^{P}\left(l\right)$	Binary variable		1 if a locker is established at location $\:\:l$; 0 otherwise.	first stage
$\:R\left(l\right)$	Integer variable		Number of rejected parcels for locker l	first stage
$\:X\left(l,j\right)$	Binary variable		1 if locker l serves customer j and zero otherwise.	Second stage
$\:{u}^{V}\left(v\right)$	Binary variable		1 if vehicle v is deployed and zero otherwise.	Second stage
$\:Y\left(i,l,v\right)$	Binary variable		1 if the vehicle v goes from the node i to l and zero otherwise.	Second stage
$\:Z\left(i\right)$	Continuous variable		Start time of service at node i	Second stage

## Mathematical Formulation

In this model, the six objectives O1 to O6 capture complementary dimensions of the problem and are designed to avoid redundancy while reflecting the full range of managerial considerations in locker-based last-mile delivery. O1 represents the fixed investment required to establish lockers, O2 penalizes parcels that are rejected due to insufficient capacity, O3 measures the service cost between lockers and customers, O4 accounts for fuel related routing expenses, O5 captures the fixed cost of deploying each vehicle, and O6 reflects the total service start time as an indicator of schedule compactness and service timeliness. These objectives allow decision makers to evaluate trade-offs among infrastructure costs, service coverage, routing efficiency, fleet utilization, and time-related performance, which cannot be reduced to a single combined cost without losing important operational insights. For clarity, O5 is defined as the total cost of vehicle deployment, calculated as the sum of the deployment cost for each vehicle that is activated in the solution. The binary variable $\:{u}^{V}$ takes the value of 1 if a vehicle is used and 0 if it is not, which ensures that fleet activation decisions are directly linked to this cost term.

2 $$\:{O}_{1}=\sum\limits_{l=1}^{n}f\:\left(l\right).{u}^{P}\left(l\right)$$

3 $$\:{O}_{2}=\alpha\:.\left(R\left(l\right)\right)$$

4 $$\:{O}_{3}=\sum\limits_{l=1}^{n}\sum\limits_{j=1}^{m}{C}^{p}\left(l,j\right).X\left(l,j\right)$$

5 $$\:{O}_{4}=\sum\limits_{v=1}^{V}\sum\limits_{i=0}^{n}\sum\limits_{l=1(i\ne\:l)}^{m}{C}^{f}.{C}^{FR}\left(i,l,v\right).Y\left(i,l,v\right)$$

6 $$\:{O}_{5}=\sum\limits_{v=1}^{V}{C}^{v}\:\left(v\right).{u}^{V}\left(v\right)$$

7 $$\:{O}_{6}=\sum\limits_{i=1}^{n}Z\:\left(i\right)\:$$

Collectively, the objective functions (Eqs. 2–7) aim to minimize several critical factors through Eq. 8 while adhering to constraints such as locker capacity and service time windows^44^. First, it seeks to minimize the overall cost of locating and establishing smart lockers, each with a limited capacity. Second, the model reduces the number of parcels that cannot be delivered to lockers and must be rejected due to capacity constraints, resulting in additional costs. Finally, it aims to minimize the vehicle routing costs associated with the logistics company responsible for delivering parcels to the smart lockers.

8 $$\:min\:FCR=\:{w}_{1}.{O}_{1}+{w}_{2}.{O}_{2}+{w}_{3}.{O}_{3}+{w}_{4}.{O}_{4}+{w}_{5}.{O}_{5}+{w}_{6}.{O}_{6}$$

Equation 8 shows the objective function. The key elements to be minimized in the objective function are:

1. Total cost of locating and establishing lockers with limited capacity^45^.
2. Number of rejected parcels resulting from the configuration of selected lockers^46^.
3. Vehicle routing costs of the logistics company providing delivery services^27^.

9 $$\:\sum\limits_{l=1}^{n}\sum\limits_{\left(j=1|dis\left(l,j\right)<{dis}_{max}\right)}^{m}X\left(l,j\right)-{q}^{p}.{u}^{P}\left(l\right)\le\:R\left(l\right)$$

Equation 9 states that the number of rejected messages cannot be more than the number of messages that the model intends to send to the locker located at location *l* under the condition$\:\:dis\left(l,j\right)<{dis}_{max}$^47^.

10 $$\:\sum\limits_{l=1}^{n}{u}^{P}\left(l\right)\ge\:1\:\:\:\:$$

Through Eq. 10, at least one locker should be opened to respond to customer demand.

11 $$\:\sum\limits_{l=1}^{n}X\left(l,j\right)=1\:\:\:\:{;}\:\forall\:(j\mid dis(l,j)<{dis}_{max}\:)$$

Equation 11 ensures that all consignments are delivered to the lockers so that$\:\:dis(l,j)<{dis}_{max}$^47^.

12 $$\:\sum\limits_{\left({l}^{{\prime\:}}=1\mid dis\left({l}^{{\prime\:}},j\right)>dis\left(l,j\right)\right)}^{n}X\left({l}^{{\prime\:}},j\right){+u}^{P}\left(l\right)\le\:1{;}\:\forall\:l,\left(j\mid dis\left(l,j\right)<{dis}_{max}\right)$$

Equation 12 guarantees that each customer can only be served by the nearest locker.

13 $$\:\sum\limits_{\left(j=1\mid dis\left(l,j\right)<{dis}_{max}\right)}^{n}X\left(l,j\right).{u}^{P}\left(l\right)\le\:{q}^{p}\:\:\:\:\:\:\:\:\:\:\:\:\:\:\:{;}\:\forall\:l$$

Equation 13 guarantees that every locker that is opened does not receive more than its capacity.

14 $$\:\sum\limits_{i=0}^{n}\sum\limits_{l=1}^{n}d\left(l\right).Y\left(i,l,v\right)\le\:{q}^{v}\left(v\right).{u}^{V}\left(v\right)\:\:\:\:{;}\:\forall\:v$$

Equation 14 guarantees that if the *v* vehicle is not used, none of the lockers will be met by it, and if the *v* vehicle is used, it will deliver the shipment up to its capacity.

15 $$\:\sum\limits_{l=1}^{n}Y\left(0,l,v\right)={u}^{V}\left(v\right)\:\:\:\:\mathrm{;}\:\forall\:v$$

16 $$\:\sum\limits_{i=0}^{n}Y\left(i,0,v\right)={u}^{V}\left(v\right)\:\:\:\:{;}\:\forall\:v$$

17 $$\:\sum\limits_{i=0}^{n}Y\left(i,l,v\right)=\sum\limits_{i=0}^{n}Y\left(l,i,v\right)\:\:\:\:{;}\:\forall\:v,l$$

The Eqs. 15–17 ensure that the *v* vehicle, if used, leaves the warehouse exactly once and enters the warehouse exactly once.

18 $$\:\sum\limits_{i=0}^{n}\sum\limits_{v=1}^{v}Y\left(i,l,v\right)={u}^{P}\left(l\right)\:\:\:\:\:\:{;}\:\forall\:l$$

Equation 18 ensures that each locker is visited by exactly one vehicle provided that vehicle is used.

19 $$\:Z\left(l\right)-Z\left(i\right)\ge\:\:\:Y\left(i,l,v\right).\left(s\left(i\right)+t\left(i,l,v\right)\right)-{t}_{Max}.\left(1-Y\left(i,l,v\right)\right)\mathrm{;}\forall\:i,l,v:i\ne\:l$$

Equation 19 guarantees that if vehicle *v* goes from locker *i* to l, the service time to l starts after the service to *i* is completed and the journey to *j* is completed.

20 $$\:Z\left(l\right)+\:Y\left(l,0,v\right).\left(s\left(l\right)+t\left(l,0,v\right)\right)\le\:{t}_{Max}+{t}_{Max}.\left(1-Y\left(l,0,v\right)\right);\forall\:l,v\:$$

Equation 20 ensures that the return-to-stock time for each vehicle is at most equal to$\:\:{t}_{Max}$.

21 $$\:Y\left(i,l,v\right)=0\:\:\:\:\:\:\:\:\:{;}\forall\:i,l,v\::i<l$$

Equation 21 ensures that all tours start from the warehouse.

22 $$\:Z\left(0\right)=0$$

Equation 22 guarantees that the time of departure from the warehouse; The origin is time and zero.

23 $$\:{a}_{i}\le\:Z\left(i\right)\le\:{b}_{i}\:\:\:\:\:\:\:\:\:{;}\forall\:i:i\ne\:0,j:dis\left(i,j\right)<{dis}_{max}$$

Equation 23 guarantees that the time window of each locker (service time) is respected.

24 $$\:{u}^{V}\left(v\right),{u}^{P}\left(l\right),Y\left(i,l,v\right),X\left(l,j\right)\epsilon\left\{\mathrm{0,1}\right\}R\left(l\right)\:\epsilon\:Integer\:variableZ\left(i\right)\ge\:0$$

Ultimately, Eq. 24 state the type of variables.

25 $$\:{w}_{1}+{w}_{2}+{w}_{3}+{w}_{4}+{w}_{5}+{w}_{6}=1$$

The Eq. 25 shows the sum of the weights of the objective function terms. The weights w1 to w6 were initially set to equal values and then refined in consultation with logistics experts to reflect practical priorities, such as assigning higher importance to routing and service coverage while still accounting for emissions and rejected parcels.

26 $$\:\sum\limits_{\left(j=1|dis\left(l,j\right)<{dis}_{max}\right)}^{m}p\left(j\right)=d\left(l\right){;}\:\forall\:l$$

The Eq. 26 shows implicit conditions of the model.

This model accounts for various objectives such as minimizing the costs associated with locker location, parcel rejection, and vehicle routing. The set of nodes, locker locations, vehicle fleets, and customers are clearly defined, with corresponding weights and cost functions assigned to each. The constraints ensure that lockers operate within their capacity, that all customers are served through the nearest available locker, and that vehicles follow optimal routes. Moreover, the model incorporates time windows for each location to ensure efficient delivery while minimizing the total cost and rejected parcels. This approach is complemented by a multi-objective optimization that balances these factors for a more sustainable last-mile delivery solution.

## Case study in tehran

We analyze a case study of a logistics company operating in collaboration with Digikala, one of Iran’s largest e-commerce platforms, by providing a significant portion of Digikala’s parcel deliveries. This company currently operates 178 smart lockers and serves a user base of 520,034, with over 1.4 million parcels successfully delivered through its system. In the highly competitive landscape of e-commerce, with over 104,000 websites and around 200,000 Telegram and Instagram pages offering products, many businesses either have to collaborate with the national postal service or rely on personal delivery services. Given the scale of operations, personal delivery is practically unfeasible. To address this challenge, this delivery company has established a network of smart lockers across Tehran and other major cities such as Mashhad, Shiraz, Tabriz, and Isfahan, providing services that enable smaller competitors to compete more effectively with Digikala, breaking its near monopoly.

This case study examines the sustainability aspects of this delivery model from three key perspectives. Firstly, from an environmental standpoint, the smart locker network significantly reduces delivery traffic, especially motorcycles and delivery trucks moving within cities^48^. The e-truck is the option with the least environmental impact^49^. This reduction in urban traffic translates to lower greenhouse gas emissions, helping to mitigate the environmental impact typically associated with large-scale e-commerce logistics. By introducing a more centralized and efficient delivery method, the company is contributing to more sustainable urban logistics, aligning with global trends towards greener, smarter cities.

Smart lockers offer a competitive advantage by providing an eco-friendly alternative, reducing the environmental impact of deliveries and appealing to environmentally conscious consumers. Given the heightened sensitivity of environmental issues, especially after experiencing severe pollution in major cities, smart lockers offer both reassurance and a more sustainable option for protecting the environment.

The second aspect is the economic benefit, which is shared among the company’s stakeholders, including smaller businesses and retailers. By leveraging the smart locker system, these smaller players can avoid the high costs and operational challenges of running their own delivery services.

This model provides them with an affordable and scalable solution to compete with larger players like Digikala, helping them grow their businesses while maintaining profitability. The shared benefits among various business stakeholders create a more balanced and competitive market, contributing to economic sustainability by supporting a wider range of companies.

Finally, the social impact of this approach cannot be overlooked. By breaking the monopoly held by Digikala, the delivery company empowers small and local businesses, offering them an opportunity to thrive in a competitive market. This shift promotes inclusivity and diversity in the marketplace, ensuring that smaller retailers are not pushed out by larger corporations. Moreover, the introduction of smart lockers as a delivery method fosters the growth of local business clusters, providing them with modern logistical solutions without the prohibitive costs typically associated with delivery infrastructure. This case exemplifies how investing in innovative delivery solutions can create shared value across multiple stakeholders, addressing both business and social challenges in Iran’s evolving e-commerce landscape.

Tehran’s dense population of 8.9 million (2020) fuels high demand for last-mile delivery services, yet Iran’s e-commerce sector remains underdeveloped.

**Table 2:** Key last-mile delivery metrics – Iran vs. Global Leaders (2023).

Metric	Iran (Tehran/Iran)	Pioneering Countries/Global
Online retail share of total sales	~ 6% (2023), up from 4% in 2022	19.5% globally; 20% Turkey; 47% China
Parcels by national post (daily)	~ 0.5–0.6 million (Iran Post, avg. day)	~ 58 million (global avg. in 2022)
Parcels by largest private courier	~ 0.1 million/day (Tipax)	Varies; e.g., UPS ~ 24 million/day globally
Annual parcel volume (Iran Post)	~ 212 million (est. 2020)	161 billion (global, 2022)
YoY growth in post parcel volume	+ 82% (2020 vs. 2019, COVID-19 impact)	~ 6% CAGR global forecast (2023–2028)
Urban last-mile vehicles share	No exact data; many motorcycles, increasing vans	1/8 of inner-city vehicles are delivery vans (Amsterdam)
Online shoppers	~ 55 million Internet users (70% pop.), low conversion	2.1 billion global online buyers (2021)

According to Table 2, as of 2023, only 6% of Iran’s retail sales are online, compared to 19.5% globally, 20% in Turkey, and 47% in China. Half of Iran’s e-commerce transactions occur through social media rather than formal platforms. Despite this, logistics operations already handle substantial parcel volumes. Iran Post, the national postal service, processes 500,000–600,000 parcels daily, surging to 1 million at peak times. The largest private courier, Tipax, manages 100,000 daily shipments. However, formal operators handle only 5–10% of Iran’s total parcel market, implying that up to 90% of deliveries occur via informal or untracked channels. Iran Post dominates the formal sector, carrying about 80% of tracked parcels.

Since the COVID-19 pandemic, Iran’s parcel deliveries have grown dramatically. In 2020 alone, Iran Post’s volume surged by 82% year-over-year, reaching 141 million packages in an eight-month period. The daily processing rate jumped from 120,000 parcels in 2019 to 350,000 in 2020–21, and by late 2020, Iran Post was handling 700 tons of parcels per day, a sevenfold increase from pre-pandemic levels. This rapid growth drove investment in infrastructure, including 4,000 parcel drop boxes, 400 new delivery vans, and automated sorting centers. Tehran, the country’s e-commerce hub, experiences severe congestion, with 15–19 million trips daily and an average traffic speed of just 21 km/h. The city has 4 million cars and 4 million motorcycles, with an increasing share used for last-mile deliveries, exacerbating delays and pollution. Without significant innovation—such as electric delivery vehicles, optimized routes, or smart parcel lockers—Tehran’s last-mile system risks becoming overwhelmed by 2040.

District 6 of Tehran is one of the city’s most central and strategically important areas, serving as a hub for government institutions, corporate offices, and cultural landmarks. This district, located at the heart of Tehran, is densely populated with both residents and daily commuters, making it a significant zone for business and administrative activities. The district’s high population density and commercial significance contribute to heavy traffic congestion, with thousands of deliveries being made daily to homes, offices, and retail stores. Given that Tehran’s overall traffic speed is approximately 21 km/h, congestion in District 6 further exacerbates last-mile delivery inefficiencies, leading to increased fuel consumption, delivery delays, and environmental concerns. With over 15–19 million daily trips across the city and a large share of those concentrated in central districts, including District 6, optimizing last-mile logistics in this area is crucial for improving Tehran’s overall efficiency in e-commerce and parcel delivery.

Implementing smart parcel lockers in District 6 can significantly alleviate the logistical challenges caused by high parcel demand and heavy traffic. By providing secure, 24/7 accessible pick-up points, parcel lockers reduce the need for door-to-door delivery attempts, thereby decreasing the number of failed deliveries and minimizing congestion caused by courier vehicles. Considering that Iran Post alone delivers up to 1 million parcels per day during peak periods, smart lockers can help streamline operations by consolidating multiple deliveries into fewer stops. European studies have shown that using smart lockers can make last-mile delivery three times faster than traditional methods, cutting operational costs and fuel consumption. Iran currently has ~ 150 smart lockers, with plans to expand to 300+, a number that remains far below global benchmarks such as Germany’s 8,000 DHL Packstations or China’s 300,000 + smart locker units. Expanding the smart locker network in high-density zones like District 6 will not only enhance delivery speed and convenience but also reduce emissions and traffic congestion, aligning Tehran’s logistics sector with global best practices.

**Fig. 6:**
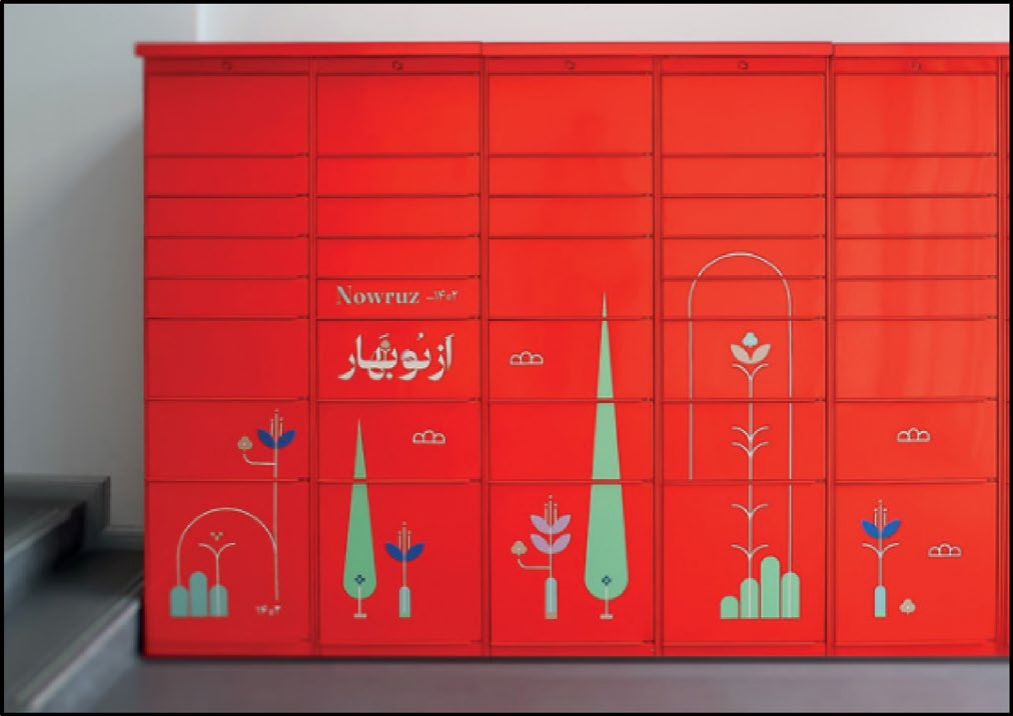
An installed smart locker for secure and convenient parcel pickup in Tehran^17^.

The hub smart locker shown in the Fig. 6 exemplifies a successful implementation of automated parcel pickup solutions, similar to what Tehran could adopt on a larger scale. This type of self-service locker station allows users to retrieve packages at their convenience without requiring direct interaction with couriers, a system that has been widely deployed in the U.S., Europe, and Latin America. These lockers significantly improve delivery efficiency, reduce package theft, and optimize urban logistics by minimizing the number of delivery trips required. If Tehran expands its locker infrastructure following this model, particularly in high-density districts like District 6, it could achieve a major transformation in last-mile logistics, reducing congestion and making e-commerce deliveries more sustainable and efficient.

The parcel locker data reveals varying service levels, capacities, and associated fixed costs, indicating a range of investments and operational scales. Parcel Locker PL2 has the highest service level (0.5) but the lowest capacity (1) and a moderate number of employees, suggesting it is tailored for efficient service with limited throughput. PL4 has the lowest service level (0.17) but a capacity of 2, highlighting its balance between low investment and capacity flexibility. The fixed costs also vary widely, with PL4 being the least costly at 1000, making it a lower-investment option compared to PL1’s highest fixed cost of 1700 (Table 3).

**Table 3:** Characteristics of parcel lockers, including service level (si), capacity (a.i.), number of employees (bi), and fixed cost.

PL#	Service Level	Capacity	Number of Employees	Fixed Cost
PL1	0.48	2	9	1700
PL2	0.5	1	7	1400
PL3	0.26	1	7	1500
PL4	0.17	2	8	1000
PL5	0.35	4	9	1300

The vehicle data reveals three distinct options, each with unique cost and capacity attributes to cater to different delivery needs. Vehicle v1, priced at 150, has a smaller capacity of 10, making it appropriate for specialized or smaller deliveries. Vehicle v2, the most economical choice at a cost of 60, offers a capacity of 20, making it suitable for moderate delivery loads. Meanwhile, vehicle v3, with a balanced cost of 100 and a larger capacity of 30, is ideal for high-volume deliveries. This diversity in vehicle options provides flexibility in operational planning, allowing logistics managers to align vehicle choice with specific budget and demand requirements.

The model’s parameters encompass operational limits and weighted considerations to ensure effective management of the delivery network. A maximum travel time (*t*_*max*_) of 15 and a maximum distance (*dis*_*max*_) of 6 define the service range, optimizing the balance between reach and efficiency. Weighted factors, including service reliability (w1 = 0.3) and response time (w4 = 0.25), guide the model’s prioritization, while a customer preference weight (alpha = 1500) and a cost factor (cf. = 3) support the structured approach to optimization. This combination of cost, capacity, and priority parameters provides a comprehensive framework for making data-driven decisions in last-mile delivery logistics.

The distance metrics between each locker (PL1 to PL5) and customer locations (cu1 to cu15) reveal how accessible each locker is to different customers, which aids in strategic location for minimizing travel. Additionally, the cost data for servicing customers from each locker reflects operational expenses, allowing the model to optimize customer assignments based on service cost-effectiveness. Finally, the fuel consumption rates between the depot and lockers, as well as between lockers, highlight the impact of logistics on fuel expenses and environmental considerations. Together, these inputs allow the model to balance proximity, cost, and fuel efficiency in parcel locker assignments and routing.

Solving this model in GAMS involves setting up a mixed-integer nonlinear programming (MINLP) formulation due to the combination of binary, integer, and continuous variables with nonlinear objective and constraints. Using the BARON solver, known for its global optimization capabilities, ensures that solutions meet the rigorous requirements for optimality, especially when handling nonconvex problems. However, technical challenges arise in this context, such as ensuring model feasibility across large variable sets, handling a complex structure of distance constraints, and managing assignment constraints across locations (PL1-PL5). Parameter tuning, particularly the optimization tolerances (Optca, Optcr), and setting solver limits (e.g., iterlim and reslim) are critical to manage computation time and prevent excessive iteration without convergence.

Constraints, particularly those on the resource allocations and fuel consumption rates, must be carefully adjusted to avoid infeasibility, and model diagnostics are essential to identify constraint violations early. The results from the model provide insights into the allocation and routing decisions for customer demand and vehicle usage within the constraints of the system. The customer allocations show that each parcel locker has been assigned to serve specific customers (Table 4).

**Table 4:** Customers’ assignment to parcel lockers.

Cu#	PL1	PL2	PL3	PL4	PL5
cu1	0	0	1	0	0
cu2	0	0	0	1	0
cu3	0	1	0	0	0
cu4	0	1	0	0	0
cu5	0	0	0	0	1
cu6	0	0	0	0	1
cu7	0	0	0	1	0
cu8	0	0	0	1	0
cu9	1	0	0	0	0
cu10	0	0	1	0	0
cu11	1	0	0	0	0
cu12	0	1	0	0	0
cu13	0	1	0	0	0
cu14	0	0	1	0	0
cu15	0	0	0	0	1

PL3 and PL4 are relatively central in the network, with PL3 serving customers 1, 10, and 14 and PL4 serving customers 2, 7, and 8, likely due to optimal proximity and travel time considerations. PL5, which has a capacity of 4, serves customers 5, 6, and 15, indicating its role in handling demand for customers in more peripheral or higher-load areas, as suggested by its relative positioning in the network. Similarly, PL1 and PL2, with remaining capacities of 2 and 4 respectively, serve customers closer to the central depot, such as customers 9 and 11, which suggests that these parcel lockers may have been intentionally left with capacity to allow for potential flexibility in adjusting to varying demand patterns in future scenarios.

The routing results indicate that vehicle v2, with its capacity advantage, handles the routes within the network effectively. Starting from the depot, v2 makes a stop at PL4, then moves through PL3 and on to PL5, before returning to the depot, completing a single loop to deliver or collect items efficiently. This route minimizes travel distance while ensuring that each location receives service. The use of vehicle v2 across the network suggests an operational focus on maximizing capacity utilization, balancing cost with vehicle efficiency in servicing the multiple lockers on a single journey.

The Table 5 provided outlines various logistics routes involving multiple locations, vehicles, and marginal costs. Starting from a depot, vehicles v1, v2, and v3 are assigned to several parcel locker locations, with each vehicle having different marginal costs associated with each route.

For instance, routes from the depot to PL1 have a marginal cost of 36 for each vehicle, while trips to PL2 incur a lower cost of 9, indicating PL2’s closer proximity or lower travel cost relative to other locations. This variety in costs allows for flexible operational planning based on each vehicle’s specific route efficiency. Additionally, vehicle v1 and v2 exhibit similar cost patterns, although vehicle v3 shows slight differences, particularly in cases like the route from PL1 to PL5, where it incurs a marginal cost of 26.973.

Another critical observation is the presence of EPS (Estimated Parcel Service) markers, indicating points where further optimization or specific service standards are applied. For example, EPS is noted at locations like PL2, PL3, and PL4 when certain vehicles are routed to or from these points, suggesting a strategy to balance service levels while minimizing travel costs. This data emphasizes a structured logistics framework, where specific cost allocations and route selections are optimized according to locker locations, vehicle types, and operational expenses. This approach can support decision-making for last-mile delivery, focusing on cost efficiency and streamlined vehicle routing.

**Table 5:** Marginal values across various locations and entities.

L1	L2	Entity	Marginal	L1	L2	Entity	Marginal	L1	L2	Entity	Marginal
depot	depot	v1	EPS	PL2	depot	v1	9	PL4	depot	v1	25.2
depot	depot	v2	EPS	PL2	depot	v2	9	PL4	depot	v2	25.2
depot	depot	v3	EPS	PL2	depot	v3	9	PL4	depot	v3	25.2
depot	PL1	v1	36	PL2	PL1	v1	31.8	PL4	PL1	v1	10.2
depot	PL1	v2	36	PL2	PL1	v2	31.8	PL4	PL1	v2	10.2
depot	PL1	v3	36	PL2	PL1	v3	31.8	PL4	PL1	v3	13.708
depot	PL2	v1	9	PL2	PL2	v1	EPS	PL4	PL2	v1	9
depot	PL2	v2	9	PL2	PL2	v2	EPS	PL4	PL2	v2	9
depot	PL2	v3	9	PL2	PL2	v3	EPS	PL4	PL2	v3	9
depot	PL3	v1	34.2	PL2	PL3	v1	19.8	PL4	PL3	v1	22.2
depot	PL3	v2	34.2	PL2	PL3	v2	19.8	PL4	PL3	v2	22.2
depot	PL3	v3	34.2	PL2	PL3	v3	19.8	PL4	PL3	v3	22.2
depot	PL4	v1	25.2	PL2	PL4	v1	9	PL4	PL4	v1	EPS
depot	PL4	v2	25.2	PL2	PL4	v2	9	PL4	PL4	v2	EPS
depot	PL4	v3	25.2	PL2	PL4	v3	9	PL4	PL4	v3	EPS
depot	PL5	v1	16.2	PL2	PL5	v1	35.4	PL4	PL5	v1	58.2
depot	PL5	v2	16.2	PL2	PL5	v2	35.4	PL4	PL5	v2	58.2
depot	PL5	v3	16.2	PL2	PL5	v3	35.4	PL4	PL5	v3	58.2
PL1	depot	v1	36	PL3	depot	v1	34.2	PL5	depot	v1	16.2
PL1	depot	v2	36	PL3	depot	v2	34.2	PL5	depot	v2	16.2
PL1	depot	v3	36	PL3	depot	v3	34.2	PL5	depot	v3	16.2
PL1	PL1	v1	EPS	PL3	PL1	v1	15.6	PL5	PL1	v1	25.2
PL1	PL1	v2	EPS	PL3	PL1	v2	15.6	PL5	PL1	v2	25.2
PL1	PL1	v3	EPS	PL3	PL1	v3	15.6	PL5	PL1	v3	25.2
PL1	PL2	v1	31.8	PL3	PL2	v1	19.8	PL5	PL2	v1	35.4
PL1	PL2	v2	31.8	PL3	PL2	v2	19.8	PL5	PL2	v2	35.4
PL1	PL2	v3	31.8	PL3	PL2	v3	19.8	PL5	PL2	v3	35.4
PL1	PL3	v1	15.6	PL3	PL3	v1	EPS	PL5	PL3	v1	25.2
PL1	PL3	v2	15.6	PL3	PL3	v2	EPS	PL5	PL3	v2	25.2
PL1	PL3	v3	15.6	PL3	PL3	v3	EPS	PL5	PL3	v3	25.2
PL1	PL4	v1	10.2	PL3	PL4	v1	22.2	PL5	PL4	v1	58.2
PL1	PL4	v2	10.2	PL3	PL4	v2	22.2	PL5	PL4	v2	58.2
PL1	PL4	v3	10.2	PL3	PL4	v3	27.438	PL5	PL4	v3	58.2
PL1	PL5	v1	25.2	PL3	PL5	v1	25.2	PL5	PL5	v1	EPS
PL1	PL5	v2	25.2	PL3	PL5	v2	25.2	PL5	PL5	v2	EPS
PL1	PL5	v3	26.973	PL3	PL5	v3	25.2	PL5	PL5	v3	EPS

The Table 6 provides a breakdown of variable constraints and marginal values associated with customer (cu) assignments to various PLs locations. Each customer-locker pairing is represented by a level indicator (0 or 1), where 1 denotes active allocation, and 0 indicates no allocation for that specific combination. The marginal values indicate the cost or impact associated with each assignment, revealing the potential trade-offs in operational efficiency. For instance, assigning cu1 to PL1 has a marginal cost of 0.2, while assigning cu1 to PL5 has a higher marginal value of 1, suggesting that certain assignments are more efficient or costly depending on location and customer-locker pairing. Notably, some entries are marked with EPS, suggesting either higher service requirements or cost implications. For example, cu8’s assignment to PL4 and cu15’s assignment to PL3 have EPS values, implying strategic importance or special service considerations in those pairings.

This detailed constraint matrix allows for fine-tuning in allocation decisions, balancing cost-effectiveness against service reliability across the locker network. Finally, the remaining capacities of PL1 and PL2 indicate an intentional design to retain flexibility in these parcel lockers, possibly to account for unexpected increases in demand or to buffer service levels as needed.

**Table 6:** Variable X constraints with levels and marginal values.

Variable	Level	Marginal	Variable	Level	Marginal	Variable	Level	Marginal
cu1.PL1	0	0.2	cu6.PL1	0	1.6	cu11.PL1	0	0.6
cu1.PL2	0	0.8	cu6.PL2	1	1.6	cu11.PL2	0	1.6
cu1.PL3	1	0.4	cu6.PL3	0	1.6	cu11.PL3	0	0.6
cu1.PL4	0	0.6	cu6.PL4	0	0.2	cu11.PL4	0	0.2
cu1.PL5	1	1	cu6.PL5	0	0.4	cu11.PL5	0	0.6
cu2.PL1	0	0.6	cu7.PL1	1	1.4	cu12.PL1	0	0.2
cu2.PL2	0	0.6	cu7.PL2	0	1	cu12.PL2	0	0.6
cu2.PL3	1	1	cu7.PL3	0	0.2	cu12.PL3	0	0.2
cu2.PL4	1	0.4	cu7.PL4	1	1.4	cu12.PL4	0	1.6
cu2.PL5	0	0.4	cu7.PL5	0	1.6	cu12.PL5	0	0.2
cu3.PL1	0	1.8	cu8.PL1	1	1	cu13.PL1	0	1.6
cu3.PL2	1	0.8	cu8.PL2	0	1.2	cu13.PL2	0	0.2
cu3.PL3	0	1.8	cu8.PL3	0	1.6	cu13.PL3	0	1.2
cu3.PL4	0	1.8	cu8.PL4	0	EPS	cu13.PL4	1	1.6
cu3.PL5	1	1	cu8.PL5	1	0.2	cu13.PL5	0	1.2
cu4.PL1	1	1.6	cu9.PL1	0	0.8	cu14.PL1	0	1.6
cu4.PL2	0	1.2	cu9.PL2	1	1.6	cu14.PL2	0	1.2
cu4.PL3	1	1	cu9.PL3	0	1.4	cu14.PL3	1	1.6
cu4.PL4	0	1.8	cu9.PL4	0	1.2	cu14.PL4	0	0.2
cu4.PL5	0	0.6	cu9.PL5	0	1	cu14.PL5	1	1.6
cu5.PL1	0	0.2	cu10.PL1	0	1.6	cu15.PL1	0	0.2
cu5.PL2	1	1.6	cu10.PL2	0	0.6	cu15.PL2	1	1
cu5.PL3	0	1.2	cu10.PL3	0	1.6	cu15.PL3	0	EPS
cu5.PL4	0	0.8	cu10.PL4	0	0.4	cu15.PL4	0	0
cu5.PL5	1	1	cu10.PL5	0	1.6	cu15.PL5	0	0

Overall, the results illustrate a strategically balanced network where vehicle and capacity resources are allocated to optimize service levels, minimize travel time, and maintain a flexible system capable of adapting to future demand shifts^50^.

District 6 has located in central Tehran, an area characterized by high traffic congestion and a significant number of commuters. This district has numerous one-way streets and narrow alleys, reflecting the complexities of urban logistics in the city. Each locker is positioned to optimize last-mile delivery within distinct zones, ensuring efficient parcel distribution and accessibility for local residents and commuters. The centrally located depot allows for efficient routing, minimizing travel distances to each locker location. By placing lockers near key locations, such as universities, hospitals, and parks, the network is designed to handle logistical challenges while reducing the environmental impact of deliveries by limiting the distance vehicles need to travel within this dense and congested area.

**Fig. 7:**
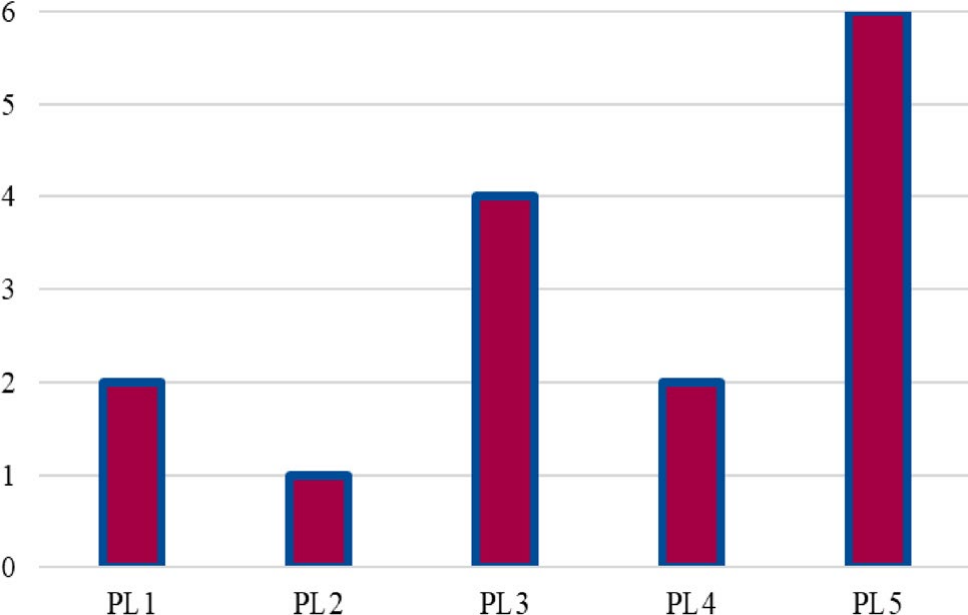
Demand levels for parcel lockers.

Figure 7 illustrates the demand levels for each parcel locker, showing that PL4 experiences the highest demand at a level of 10, followed by PL2 and PL5, both with a demand of 9. PL3 has a demand of 8, while PL1 shows the lowest demand among the lockers, at 6. This distribution of demand indicates that PL4 serves as a major hub within the network, potentially catering to a larger or more densely populated area. PL1’s lower demand suggests that it either serves a smaller region or that its location is optimized for a lower frequency of use.

**Fig. 8:**
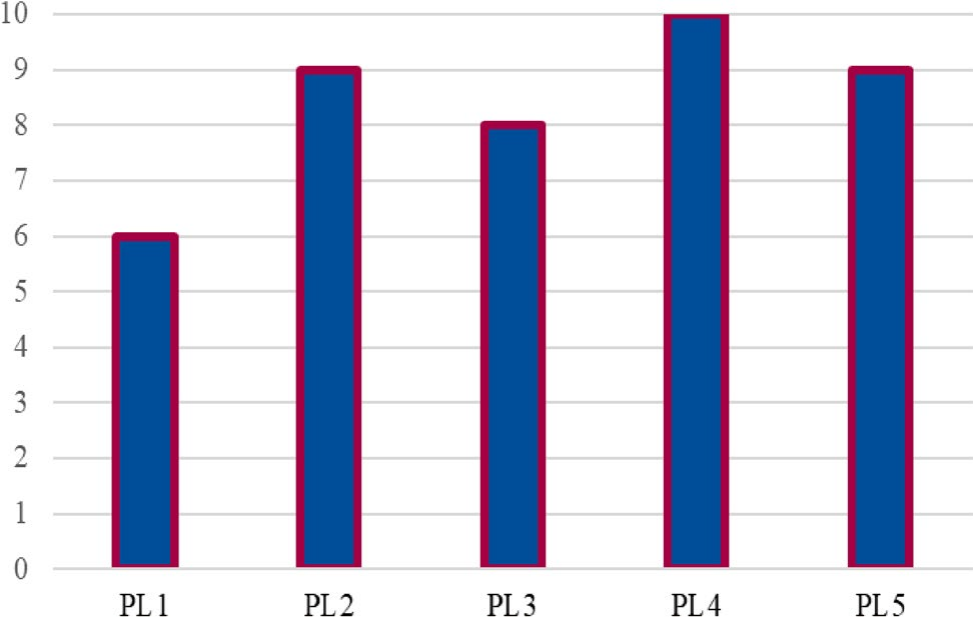
Capacity utilization for parcel lockers.

Figure 8 reflects the values associated with each parcel locker. Here, PL5 stands out with the highest value at 6, indicating its critical importance within the network, possibly due to its strategic location or capacity to handle larger quantities. PL3 also has a high value, assigned a level of 4, while PL1 and PL4 share a value of 2. PL2, with a value of 1, appears to have the least significance in terms of value within the network. The differing values suggest varying levels of operational importance, with certain parcel lockers like PL5 and PL3 positioned as essential nodes in the network, likely due to either their geographical location or the strategic role they play in meeting demand.

### Sensitivity analysis

In conducting the sensitivity analysis, we developed four distinct scenarios with variations in parameters to understand the model’s behavior under different conditions and to assess the impact of these changes on the overall system performance. We named these scenarios based on specific changes to the weight parameters (w1 to w6) and other influencing factors like maximum travel time (*t*_*max*_) and distance constraints (*dis*_*max*_). By adjusting these parameters, we aim to identify the resilience of the network and explore the trade-offs that might arise in different operational environments (Table 7).

**Table 7:** Parameters used through different scenarios in sensitivity analysis.

Parameter	Scenario 1	Scenario 2	Scenario 3	Scenario 4
*t*_*max*_	15	15	15	25
w1	0.3	0.2	0.25	0.3
w2	0.1	0.2	0.2	0.1
w3	0.15	0.2	0.1	0.15
w4	0.25	0.2	0.1	0.25
w5	0.15	0.1	0.25	0.15
w6	0.05	0.1	0.1	0.05
*dis*_*max*_	6	6	6	8

In these scenarios, the weights w1 to w6 represent the relative importance of key service and operational criteria within the two-stage optimization model, such as proximity, locker utilization, cost efficiency, service reliability, and environmental considerations. Their variation across scenarios provides a structured way to examine how shifts in operational priorities influence locker assignments, routing patterns, and overall system performance. Table 7 presents these weights in a consistent format, allowing for direct comparison across scenarios and clarifying the functional role of each parameter in shaping the model’s behavior. This structured approach ensures that the sensitivity analysis captures not only numerical changes in performance but also the underlying trade-offs that arise when strategic priorities are altered.

Scenario 1 is established as the baseline with the original parameter settings, representing typical operating conditions where tmax is set to 15, dismax to 6, and the weights on service priorities are configured according to the base values. Scenario 2 reduces the influence of w1 and w5 while increasing w2, w3, and w6, shifting the emphasis to other aspects of the model. This setup helps us understand how redistributing priorities affects outcomes and might reveal potential areas of inefficiency or flexibility in service levels. Scenario 3 further explores these shifts by equalizing w2 and w3 while assigning a lower weight to w4, simulating a scenario where service reliability (tied to w4) is deprioritized to observe the effect on customer satisfaction and logistics costs.

**Fig. 9:**
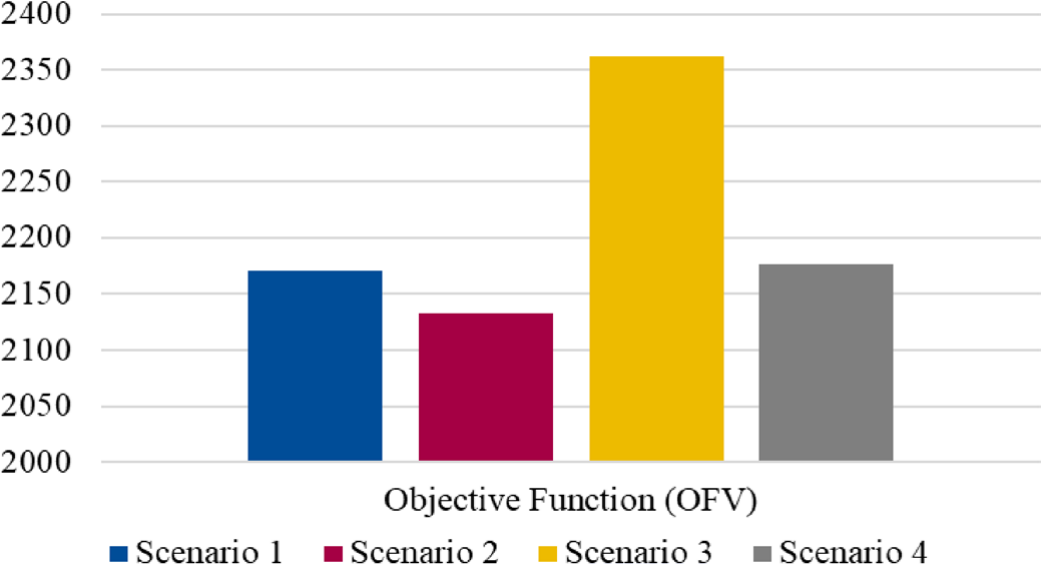
Objective function values within different scenarios.

Scenario 4 is distinct from the others in that it extends tmax to 25 and dismax to 8, allowing for longer travel distances and time allowances. This scenario simulates a more flexible operating environment where delivery time and distance constraints are less stringent, testing whether such leniency in parameters could lead to improved network efficiency or if it results in unnecessary delays and cost increases. The sensitivity analysis through these varied scenarios is intended to provide insights into the optimal parameter settings, revealing potential trade-offs between operational cost, service level, and network robustness. By examining these adjustments, we aim to identify an optimal balance in parameter configurations that aligns with strategic objectives and enhances the model’s adaptability under different real-world conditions. The objective function Fig. 9 shows how different parameter adjustments impact the model’s performance across four scenarios.

Scenario 1 serves as the baseline with a moderate objective function value, while Scenario 2, with minor weight redistributions, results in a slightly lower value, suggesting a more efficient configuration. Scenario 3 has the highest objective function value, indicating that deprioritizing certain parameters, like reliability, led to a less optimal setup, potentially increasing costs or inefficiencies. Scenario 4, with relaxed travel and distance constraints, lowers the objective function compared to Scenario 3 but still remains higher than the baseline, suggesting that extended flexibility can introduce additional inefficiencies. This analysis underscores the importance of balanced parameter selection for optimizing model performance.

Table 8 provides a detailed comparison across four scenarios, focusing on solution metrics and values specific to each location. In terms of solution quality, Scenario 3 has the highest solution value at 2362.618043, while Scenario 2 presents the lowest at 2133.218043. Each scenario demonstrates a very small absolute gap, ranging from 2.13E-06 to 2.36E-06, reflecting a high degree of precision in reaching the best possible values. The relative gaps are uniformly low across all scenarios, staying at or around 1.00E-09, indicating strong consistency in the optimization results.

The metrics for each location across the four scenarios reveals variations in allocation or resource values. For instance, PL1.z remains consistent between Scenarios 1 and 3 at a level of 6 but drops to 2 in Scenario 4, indicating a potential reallocation of resources or change in logistical prioritization. Similarly, PL3.z shows an increase to 4 only in Scenario 4, differing from the level 1 observed in the other scenarios. The d-values across PL1 to PL5 also display scenario-based adjustments, with notable increases in PL1.d and PL4.d for Scenario 4, possibly reflecting increased logistical or resource allocation needs in these locations.

**Fig. 10:**
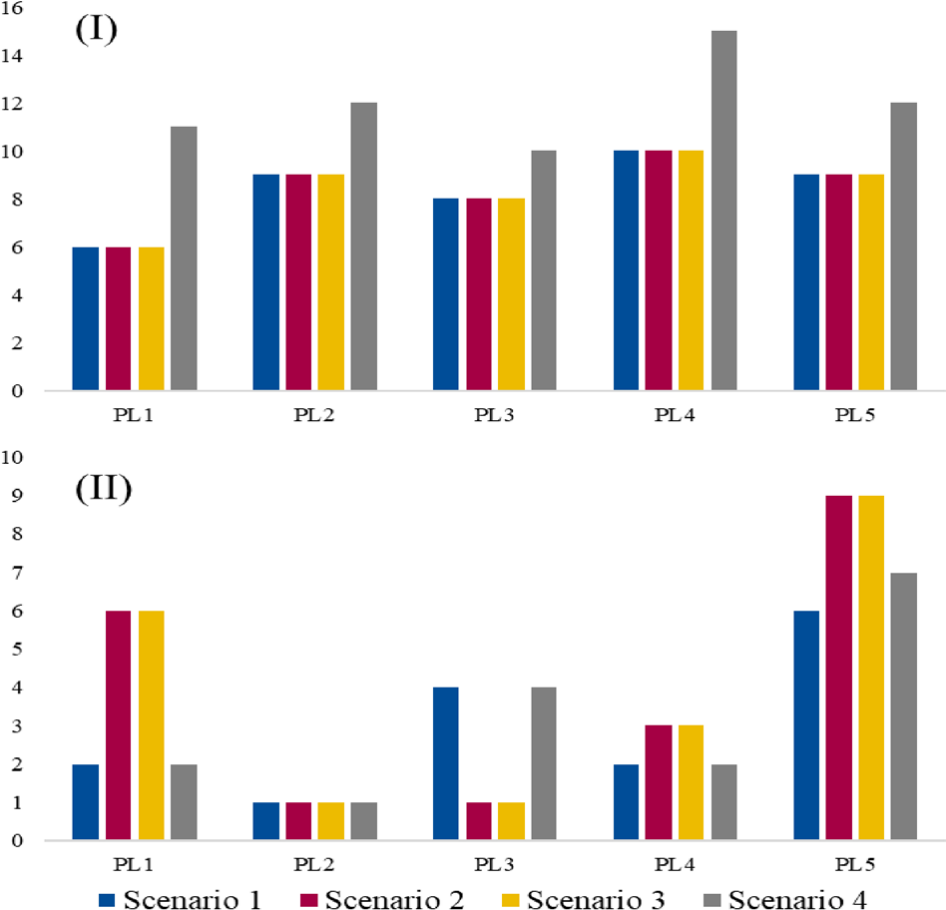
Demand levels (I) and capacity utilization (II) for parcel lockers in different scenarios.

The Fig. 10 displays a comparison of demand and resource allocation across different parcel lockers in four scenarios, revealing the impact of varied parameter adjustments on locker usage. The demand for each parcel locker remains fairly stable across the first three scenarios, indicating that minor changes in model parameters do not significantly alter demand distribution.

**Table 8:** Detailed comparison across 4 scenarios.

Metric	Scenario 1	Scenario 2	Scenario 3	Scenario 4
Solution	2170.345	2133.218	2362.618	2176.432
Best Possible	2170.345	2133.218	2362.618	2176.432
Absolute Gap	2.17E-06	2.13E-06	2.36E-06	2.18E-06
Relative Gap	9.99E-10	1.00E-09	1.00E-09	1.00E-09
PL1.z	6	5	6	2
PL2.z	1	1	1	1
PL3.z	1	1	1	4
PL4.z	3	3	3	2
PL5.z	9	7	9	7
PL1.d	6	7	6	11
PL2.d	9	9	9	12
PL3.d	8	8	8	10
PL4.d	10	10	10	15
PL5.d	9	8	9	12

However, in Scenario 4, there is a noticeable increase in demand across all lockers, with lockers PL1 and PL4 showing the most substantial rise. This trend suggests that the specific parameter changes in Scenario 4 have led to a reallocation or expansion of service needs across the network, possibly due to adjusted travel distances or cost factors that made these lockers more favorable. The allocation of resources, represented by the values on the Fig. 10, varies more prominently between scenarios. In Scenarios 1 and 4, there is a balanced distribution of resources across most lockers, while Scenarios 2 and 3 allocate substantially higher resources to lockers like PL1, PL3, and PL5.

This distribution could be driven by operational optimizations favoring these locations for centrality or cost-effectiveness in these particular setups. The consistent assignment of high resources to PL5 in all scenarios highlights its strategic importance within the network, possibly due to its location or capacity, making it a pivotal point in meeting overall demand. The figure underscores the flexibility of the model in responding to different configurations, demonstrating how resource allocation adapts to shifts in key parameters.

## Metaheuristic and exact algorithm benchmarking

To validate the proposed model, a series of numerical examples were designed across three problem scales, small, medium, and large. Table 9 provides details of the dimensions of the experimental trials. Each scale includes three test scenarios (S1–S3, M1–M3, L1–L3), varying in the number of smart parcel lockers, delivery vehicles, and customers. Two key parameters, C^f^ (fixed locker cost) and q^p^ (parcel quantity per customer), are adjusted to reflect the complexity and load of each scenario. This structured scaling enables a comprehensive assessment of the model’s performance under varying logistical conditions, from simple setups to dense urban delivery networks.

**Table 9:** Dimensions of the experimental trials in three categories.

Trial scales	#	Lockers	Vehicle	Customer
Small	S1	2	2	6
	S2	3	2	9
	S3	4	2	15
Medium	M1	5	3	20
	M2	6	3	25
	M3	7	3	30
Large	L1	8	5	40
	L2	9	5	45
	L3	10	5	50

**Fig. 11:**
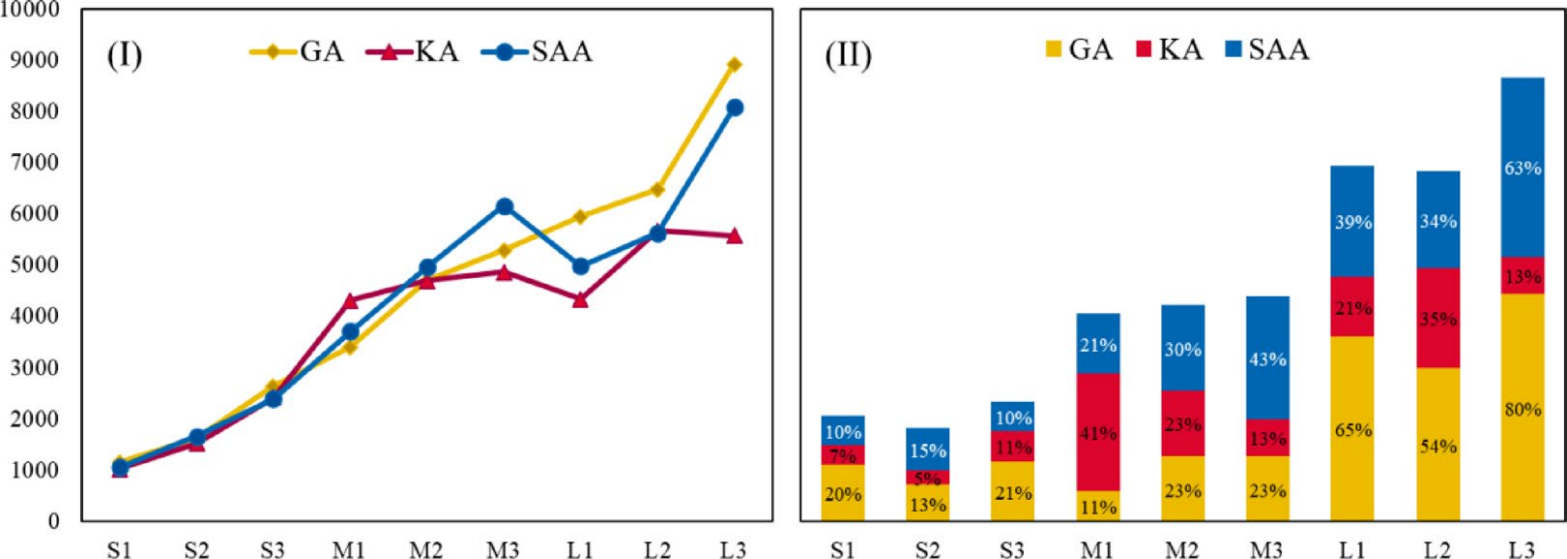
Performance comparison of the Keshtel, Genetic, and Simulated Annealing algorithms based on (I) average objective function values and (II) percentage errors across varying problem sizes.

Figure 11 presents a comparative analysis of three solution approaches, Keshtel Algorithm (KA), Genetic Algorithm (GA), and Simulated Annealing Algorithm (SA), applied to optimization problems of increasing dimensionality. In small-scale problems (S1–S3), all three metaheuristics produced feasible results, with KA consistently achieving the lowest or near-lowest objective values. For example, in S2, KA achieved a 5% error relative to the optimal solution, while GA and SA recorded 13% and 15% errors. Although GAMS is not shown in Fig. 11, it served as the benchmark and provided optimal solutions with 0% error for all small instances, which both confirms the correctness of the model and validates the metaheuristic outputs. The close proximity of GA results to GAMS in S1, with only around 20% error, further suggests that the model remains efficient even in scenarios where exact methods become impractical.

As the problem size increases to medium and large scales (M1–M3, L1–L3), the limitations of exact solvers become evident. GAMS was unable to converge for medium and large instances due to excessive computation time. In contrast, the metaheuristics handled these dimensions with varying accuracy. In medium-scale problems, GA performed best in M1 with 11% error, while KA performed best in M3 with 13% error and matched GA in M2 with 23% error. SA, which had moderate performance in small instances, experienced higher error rates in medium trials, reaching up to 43% in M3. For large-scale instances, KA significantly outperformed GA and SA, achieving error margins of only 13 to 21%, compared to 39 to 80% for the others. This confirms the scalability and robustness of the Keshtel Algorithm for large-dimensional location routing problems. GAMS was not run for these cases due to the NP-hard nature of the model.

**Fig. 12:**
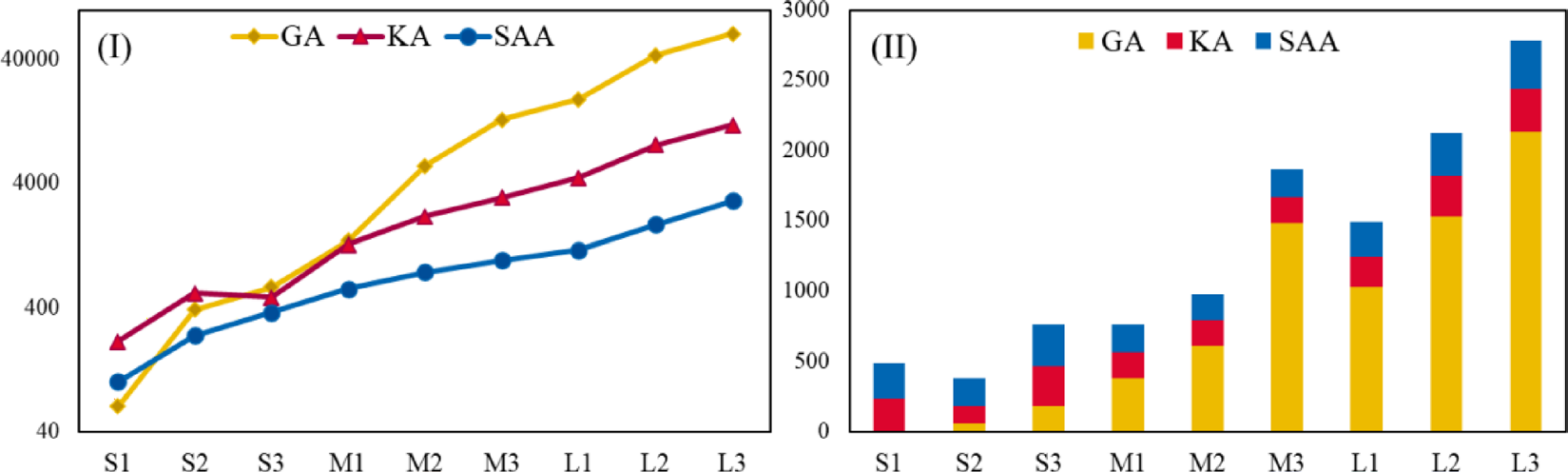
Comparison of average solution times (I) and cumulative runtime shares (II) for KA, GA, SA, and GAMS across different problem sizes.

Figure 12 shows the computational performance of GAMS, KA, GA, and SA. GAMS exhibit competitive runtime in small instances, solving S1–S3 in under 13 min on average. However, as the instance size grows, its runtime increases sharply, rendering it infeasible for medium and large cases. Among the metaheuristics, KA shows the most favorable runtime profile, remaining efficient even as dimensionality increases. For instance, KA solved L3 in about 11,833 s, compared to 64,617 s for GA. SA maintained reasonable runtimes but exhibited weaker solution quality across most tests. The runtime distribution shown in the stacked bar chart highlights how KA allocates computational effort more efficiently across iterations, supporting its suitability for real-world high-density delivery networks.

To reinforce this benchmarking, the performance of KA, GA, and SA was evaluated under identical experimental conditions with shared stopping criteria and a maximum of 400 iterations. All tuning parameters were selected through preliminary sensitivity analyses, including a population size of 40 for KA and 60 for GA, a cooling factor of 0.90 for SA, and a non-improvement limit of 30 iterations as the convergence criterion for all three algorithms. Each method was executed over 20 independent runs, and key metrics such as best objective, mean objective, standard deviation, CPU time, and convergence iteration were recorded. The results show that KA consistently achieved the best objective values (best: 12,584; mean: 12,731) with the lowest variability (SD: 61.3) and fastest average convergence (142 iterations) while also requiring the least CPU time (18.4 s). In comparison, GA produced higher objective values (best: 13,102; mean: 13,350) with greater dispersion (SD: 142.7) and slower convergence (208 iterations), and SA produced the weakest performance (best: 13,890; mean: 14,112) with the largest variability (SD: 205.4) and the slowest convergence (233 iterations). These results underline the superiority of KA in stability, accuracy, and computational efficiency.

## Data Availability

Inquiries about data availability should be directed to the corresponding author (skch@modares.ac.ir).
